# Magnetic‐Driven Viscous Mechanisms in Ultra‐Soft Magnetorheological Elastomers Offer History‐Dependent Actuation with Reprogrammability Options

**DOI:** 10.1002/advs.202506790

**Published:** 2025-08-13

**Authors:** Ernesto Gonzalez‐Saiz, Maria Luisa Lopez‐Donaire, Lucía Gutiérrez, Kostas Danas, Daniel Garcia‐Gonzalez

**Affiliations:** ^1^ Department of Continuum Mechanics and Structural Analysis Universidad Carlos III de Madrid Calle Butarque 15, Leganes 28911 Madrid Spain; ^2^ Instituto de Nanociencia y Materiales de Aragón (INMA, CSIC/UNIZAR) and CIBER‐BBN Zaragoza 50018 Spain; ^3^ LMS, CNRS, École Polytechnique Institut Polytechnique de Paris Palaiseau 91128 France

**Keywords:** constitutive model, magnetorheological elastomer, mechanical memory, resevoir computing, viscoelasticity

## Abstract

This work elucidates an important open question in the field of mechanically soft magnetorheological elastomers (MREs): how microstructural rearrangements during magnetic actuation modulate their viscoelastic behavior. Experimental assays are provided on mechanically confined and very soft MREs that, under magnetic actuation, show an order of magnitude increase in relaxation times compared to purely mechanical cases. It is demonstrated that such a modulation in the viscous response can be tuned by the amplitude and actuation rate of the magnetic stimuli, and is intrinsically linked to microstructural rearrangements of the magnetic particles. Motivated by these experimental observations, magnetic actuation protocols are conceived to enable mechanical responses in soft materials with force‐memory. Specifically, due to the magnetically induced long‐term viscous relaxation, one can induce magnetic‐driven yielding by introducing material hardening during cycling loading. This mechanical memory of the MRE can be subsequently removed by releasing the magnetic stimuli for 1 h, resetting the material performance and its microstructural state. These mechanisms are deeply understood by a combination of different experimental approaches and a new theoretical magneto‐mechanical continuum model. The results reported herein respond to unraveled fundamental questions in soft MREs, and provide a game‐changing concept for designing a new branch of soft sensor‐actuator and reservoir computing systems.

## Graphical abstract

Magnetically induced microstructural rearrangements in ultra‐soft magnetorheological elastomers modulate their viscoelasticity, enabling programmable mechanical memory. Magnetic actuation induces long‐term viscous relaxation and double‐bumping stress responses due to particle chain formation. These behaviors, captured by a new magneto‐mechanical continuum model, offer tunable actuation modes and open avenues for soft robotics and reservoir computing.

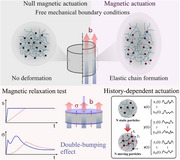

## 1 Introduction

Magnetorheological elastomers (MREs) are increasingly used by now in modern applications owing to their capability to react to external magnetic fields. The vast majority of these applications take advantage of two main features exhibited by MREs: tunable mechanical properties (stiffness, natural frequency, damping capacity, etc.), such as in vibration absorbers/isolators, sandwich beams,^[^ ^1^, ^2^ ^]^ soft robotics,^[^ ^3^, ^4^ ^]^ bio‐medicine, or industrial components;^[^ ^5^, ^6^, ^7^ ^]^ and their functional shape‐morphing characteristics, used for soft actuators,^[^ ^8^ ^]^ drug delivery systems,^[^ ^9^ ^]^ surface patterning,^[^ ^10^, ^11^ ^]^ and metastructures.^[^ ^12^, ^13^, ^14^, ^15^, ^16^ ^]^ MREs are composite materials consisting of an elastomeric matrix and magnetic particles. There are two main types of fillers that are currently used to fabricate such composites. The first type involves soft‐magnetic particles (i.e., fully energetic, with very low magnetic remanence) leading to magnetically‐soft MREs (sMREs), which are ideal for tuning their mechanical properties under magnetic actuation (e.g., magnetorheological effect^[^ ^17^, ^18^ ^]^). The second type involves hard‐magnetic particles (i.e., dissipative, with high magnetic remanence), leading to hard‐magnetic MREs (hMREs). The latter exhibit very high torque sensitivity when subjected to an external magnetic stimuli, offering the aforementioned shape‐morphing characteristics.^[^ ^19^, ^20^, ^21^ ^]^

In this work, we focus on sMREs and its connection to applications where fast mechanical responses to external magnetic loads are needed. sMREs have been active in literature for the last two decades. During this time, the large majority of studies has explored the mechanical and magnetic properties of rather stiff sMREs (E >200 kPa),^[^ ^22^, ^23^, ^24^, ^25^, ^26^, ^27^ ^]^ leaving aside the characterization of softer sMREs (E <50 kPa). Compared to the conventional stiffer sMREs, the response of soft sMREs is governed by different magnetic actuation mechanisms. As a consequence of the fairly low stiffness of the polymer matrix, large deformations are induced when magnetic stimuli are applied. This fosters potentially significant rearrangements of the particles inside the MRE.^[^ ^23^, ^28^ ^]^ In turn, due to their viscoelastic nature, soft sMREs can provide unusual time‐dependent responses,^[^ ^29^, ^30^ ^]^ reinforcing the idea of microscale particle interactions taking place while being influenced by macroscopic loading effects.

From a different viewpoint, the observed underlying mechanisms in ultra‐soft sMREs may be rationalized as similar to those observed in magnetorheological fluids (MRFs),^[^ ^31^ ^]^ since the matrix material in the second exhibits a very soft response too. Nonetheless, ultra‐soft polymers can have a substantially different mechanical response from that of a fluid, thus necessitating a careful experimental and modeling study to deeply understand the coupled magneto‐mechanical behavior occurring at the microscale of sMREs and how this affects the macroscopic response.

In this regard, the current magneto‐mechanical actuation approaches are based on the control of magnetic stimuli amplitude to modulate the deformation or forces induced within the responsive sample. Here, by exploiting novel magneto‐mechanical tests, we study and model the complex microstructural mechanisms in such ultra‐soft sMREs. Taking advantage of the strong magnetically‐induced viscous effects, we conceptualize a game‐changing actuation mode, that consists in the introduction of mechanical memory within the responsive sample in such a way that the actuating deformation or force can be modulated by the rate and number of stimuli cycles, without the need of changing the magnetic field amplitude. This allows to constrain the stimuli to a given magnitude and shape while allowing wide modulation ranges in terms of actuating forces transmitted from the sample. In addition, the magneto‐mechanically driven viscous mechanisms identified herein open new opportunities for reservoir computing.

## 2 Results

### 2.1 Viscous Relaxation Mechanisms are Strongly Modulated by Magneto‐Mechanical Interactions

**Figure 1:**
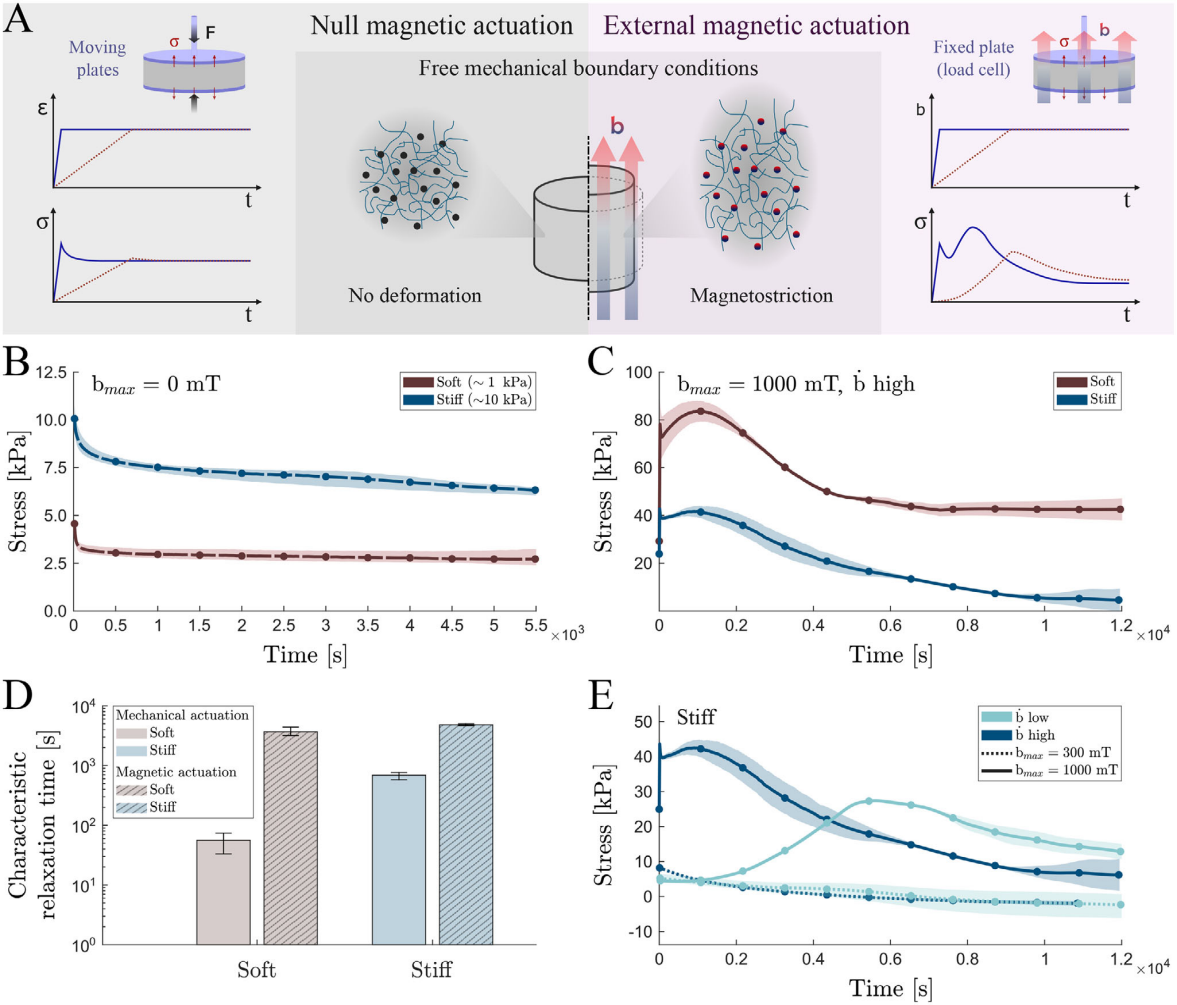
Magnetic actuation modulates viscous relaxation processes. A) Soft‐magnetic magnetorheological elastomer (sMRE) concept and scheme of the magnetostriction fundamentals based on the magnetic interactions between particles at the microstructural level. The influence of mechanical (left) and magnetic (right) loading rates on viscous relaxation mechanisms is presented, accompanied by schematics of testing protocols for evaluation of mechanical and magnetic relaxation tests. B) Purely mechanical relaxation tests on soft (≈1 kPa) and stiff (≈10 kPa) sMRE (30% CIP volume fraction) cylindrical samples that undergo a fast axial compression of 50% in absence of magnetic field. C) Comparison of stress relaxation tests applying an external magnetic ramp reaching 1000 mT in 1 s, measuring the time evolution of the axial stress on mechanically confined soft and stiff sMREs samples (30% CIP volume fraction, diameter =18 mm). D) Quantification of the representative characteristic relaxation times for the soft and stiff sMRE samples under mechanical and magnetic loading. Comparison of stress relaxation tests applying external magnetic ramps combining different magnetic field magnitudes (300 and 1000 mT) and loading rates (${\dot{b}}_{\mathrm{low}}=0.2$ mT·s^−1^ and ${\dot{b}}_{\mathrm{high}}=1000$ mT·s^−1^) on stiff sMREs samples (30% CIP volume fraction, diameter =18 mm).

Previous studies have identified two primary mechanical responses of sMREs to external magnetic actuation: magnetorheological and magnetostrictive effects.^[^ ^31^, ^33^ ^]^ Magnetorheological effects refer to material stiffening due to internal magnetic stresses, while magnetostrictive effects involve deformations and changes in geometry driven by these stresses (**Figure** **1**A). This work investigates how these responses evolve in mechanically confined samples that exhibit no overall macroscopic deformation. Specifically, magnetic stimulus generates interaction forces between the particles, which are transmitted to the polymeric matrix, leading to sample deformation (Figure 1A). In confined conditions when using stiff polymeric matrices, these forces are countered by the mechanical stiffness of the matrix and strong local mechanical stresses, minimizing substantially any potential microstructural rearrangement. In contrast, with softer matrices, internal mechanical equilibrium is achieved through significant microscopic particle rearrangement and local deformations within the matrix. Furthermore, in prior research,^[^ ^31^ ^]^ we reported preliminary findings indicating that such magnetic actuation may activate also important viscoelastic mechanisms. However, those results left an important question unanswered: in mechanically confined sMRE samples under magnetic actuation, do transient yielding behaviors depend on the actuation rate, or are we observing sharp shifts in apparent viscous relaxation instead?

Before investigating these effects, we first perform mechanical relaxation tests on two different sMRE samples under null magnetic actuation. To this end, we use a TA HR‐20 rheometer equipped with a Magneto‐Rheology Accessory (Waters TA Q600, TA Instruments, New Castle, DE, USA) (more details in Methods). These samples contain a 30 vol.% of carbonyl iron powder (CIP) and two different elastomeric matrices, hereafter referred to as soft (≈1 kPa) and stiff (≈10 kPa) (more details on manufacturing protocol in Experimental Section). The label “stiff” is used with a slight abuse of notation since it corresponds to rather soft mechanical response, much softer than most sMRE materials proposed in the literature, which exhibit moduli in the order of several tens to hundreds of kPa.^[^ ^26^ ^]^ These results are shown in Figure 1B. The tested materials present a sharp stress relaxation with time following an inverse exponential decay. The soft MRE has a characteristic relaxation time of 56 s, whereas the relaxation time of the stiff MRE is higher, i.e., 688 s.

Subsequently, we perform relaxation tests by applying a rapid magnetic actuation ramp to mechanically confined samples (see scheme in Figure 1A ‐ right). Results for both soft and stiff MREs are shown in Figure 1C. Surprisingly, the stress relaxation responses exhibit a complex non‐monotonic, nonlinear behavior, with the softer sMREs leading to higher stress values. Initially, both samples present a sharp increase in stress followed by a relaxation phase over time, similar to that observed in purely mechanical relaxation tests. However, instead of a monotonous decrease of the stress, the samples exhibit a secondary stress increase, which may be larger than the first one, followed by a significantly slower relaxation phase. More details on both short‐ and long‐term responses of these MREs can be found in Figure S1 (Supporting Information). In addition, we experimentally confirmed that these responses are a direct consequence of internal magneto‐mechanical coupling and not due to thermally‐activated mechanisms (Figure S2, Supporting Information). Overall, a comparison between the magnetic and mechanical relaxation tests reveals a remarkable increase in the apparent characteristic relaxation times, by one order of magnitude for the stiff MREs, 4810 s, and two orders of magnitude for the soft MREs, 3675 s (Figure 1D).

In order to investigate further the emergence of both short‐ and long‐term stress variations followed by stress relaxation (hereafter referred to as “double‐bumping”), we carry out additional tests varying the amplitude and rate of external magnetic actuation. These results are shown in Figure 1E for the stiff MRE and in Figure S1 (Supporting Information) for the soft MRE. The findings suggest that double‐bumping only occurs when the magnetic actuation reaches a certain threshold, in terms of amplitude and rate of application of the magnetic loading. This phenomenon may also depend on the stiffness of the polymeric matrix as well. Specifically, softer matrices facilitate microstructural particle rearrangement, which modulates the magnetic stresses and influences internal viscous relaxation. Although magnetically driven viscoelastic mechanisms are the leading hypothesis for this behavior, further experimental evidence presented below is required to fully validate this explanation.

### 2.2 Microstructural Rearrangement of Particles is Driven by Magnetic Actuation, Influenced by Viscoelasticity and Determines the Macroscale Characteristic Relaxation Times

**Figure 2:**
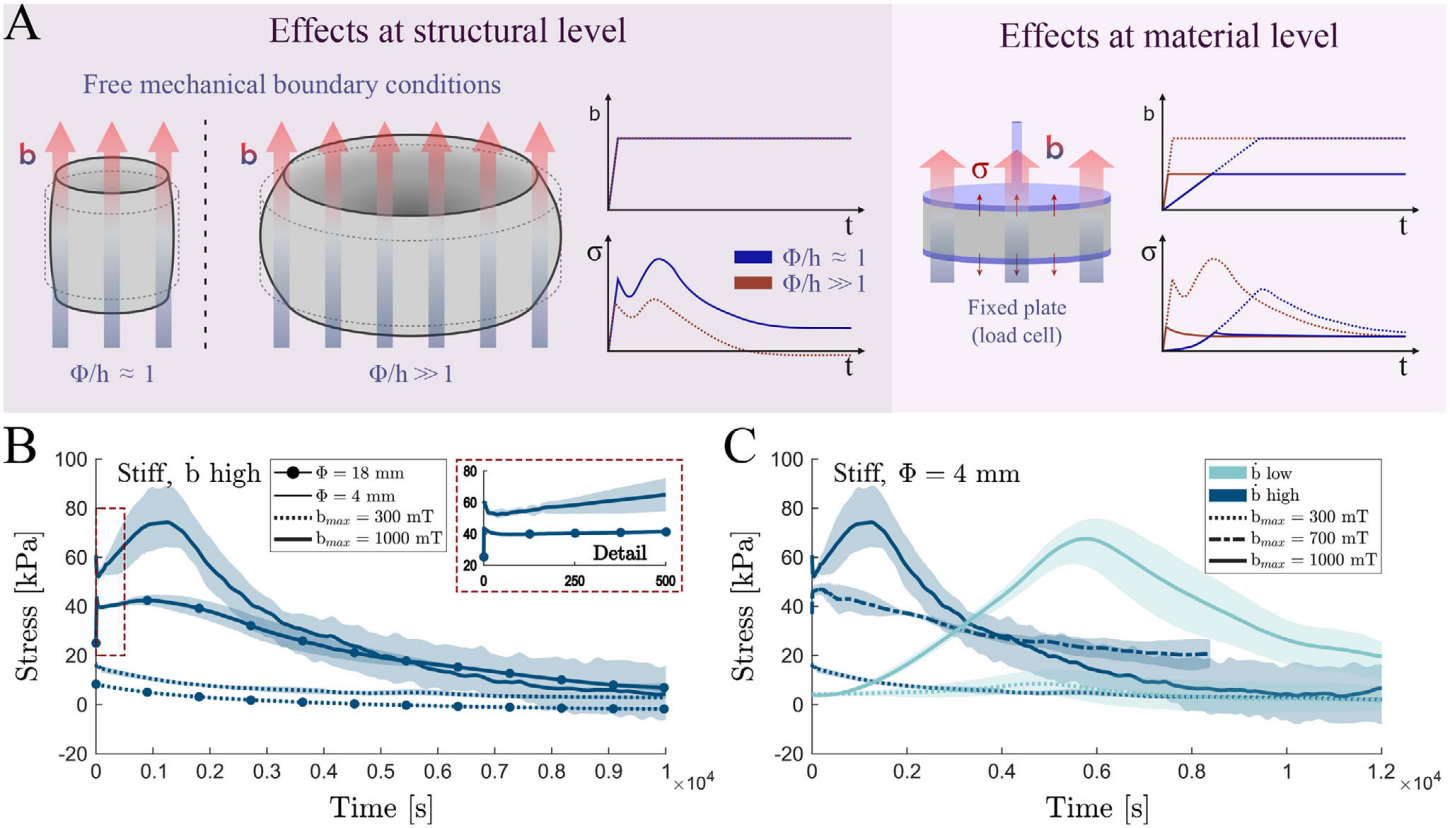
Structural effects on magnetically induced relaxation processes, and dependences on magnetic actuation characteristics. A) Scheme representing structural effects (left) affecting the magnetostriction response in soft‐magnetic magnetorheological elastomers (sMRE). The sample aspect ratio determines the magnetic fringing effects influencing the spatial concentration and distribution of magnetic fields within the sample, which modulate the magnetostriction response. Therefore, these geometrical features also modulate the relaxation response of the samples under magnetic actuation and mechanically confined boundary conditions. At the material level (right), the characteristics of the magnetic actuation (i.e., application rate and magnitude) influence the relaxation responses in sMREs. B) Comparison of stress relaxation tests applying external magnetic ramps at a high loading rate (1000 mT·s^−1^) combining different magnetic field magnitudes (300 and 1000 mT) and sample diameters (4 and 18 mm) on stiff (≈10 kPa) sMREs samples of 1 mm height (30% CIP volume fraction). C) Comparison of stress relaxation tests applying external magnetic ramps combining different magnetic field magnitudes (300, 700, and 1000 mT) and loading rates (${\dot{b}}_{\mathrm{low}}=0.2$ mT·s^−1^ and ${\dot{b}}_{\mathrm{high}}=1000$ mT·s^−1^) on stiff sMREs samples of 4 mm diameter.

The results in Figure 1 highlight complex, magnetically driven relaxation mechanisms in soft sMRE samples, revealing an unprecedented relaxation behavior defined as double‐bumping, and substantial modulation of viscous relaxation through external magnetic actuation. This response likely arises from a combination of macrostructural (structure) and microstructural (material) factors (**Figure** **2**A). Understanding the primary contributors to this behavior is essential for designing advanced materials for smart sensing and actuation. Our initial focus is to identify the underlying nature of this response: whether it is primarily structural, driven by shape‐induced magnetic effects creating heterogeneous stresses within the samples, or fundamentally microstructural. Demonstrating that this behavior has a microstructural origin would open significant opportunities for applications in multifunctional soft robotics and other innovative fields.

At the structural level, the shape and aspect ratio of the samples play a key role in determining magnetic stress distributions. Samples with diameters significantly larger than their height induce strong localized deformations due to edge effects (Figure 2A).^[^ ^33^, ^34^, ^35^ ^]^ In contrast, samples with aspect ratios close to one exhibit lower stress heterogeneity (Figure 2A). To assess these structural effects, we conducted magnetic relaxation tests on cylindrical samples with various diameter‐to‐height ratios. The results, obtained under high magnetic loading rates and two different field magnitudes, are shown in Figure 2B for the stiff sMRE samples. These findings indicate that, while the shape of the sample affects stress levels, the double‐bumping effect occurs regardless of the sample aspect ratio. This suggests that the origin of the effect lies at the material (microstructural) level, and is modulated by multiple factors such as sample geometry, matrix stiffness, magnetic field strength, and magnetic loading rate. The dependence on matrix stiffness, field magnitude, and loading rate is shown for stiff sMRE samples in Figure 2C, and for soft sMRE samples in Figure S3 (Supporting Information). These results demonstrate that an actuation threshold for the double‐bumping response exists, strongly influenced by these variables.

**Figure 3:**
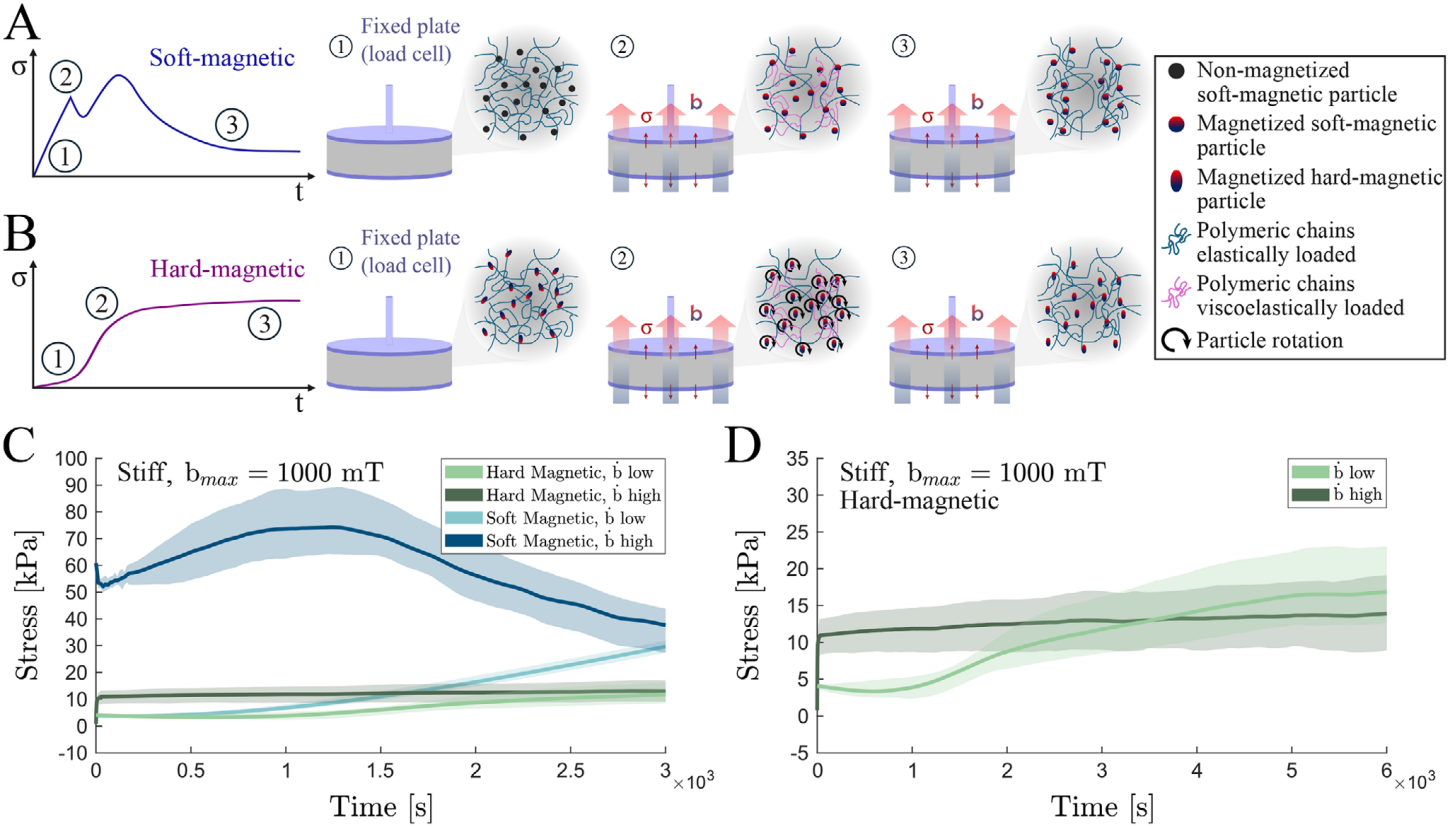
Local microstructural viscoelastic mechanisms determine macroscopic mechanical relaxation processes. A) Representative stress relaxation curve during magnetic actuation on soft‐magnetic MREs (sMREs) under mechanically confined conditions with schematic representations of the microstructural evolution during loading and relaxation. At non‐actuation conditions (1) the soft magnetic particles are isotropically distributed and present null magnetization. When an external magnetic field is applied rapidly (2), the particles magnetize introducing interactive forces and rearranging by forming chain‐like structures. This redistribution introduces local viscoelastic deformation within the polymeric chains between particles. After reaching the targeted magnetic field magnitude, if this is kept constant, the viscoelastically loaded chains relax over time enhancing the particle aligning and releasing viscous stress contributions (3). B) Representative stress relaxation curve during magnetic actuation on hard‐magnetic MREs (hMREs). In these materials, the particles present residual magnetization and, under magnetic actuation, they tend to rotate aligning along the actuating field direction. Similar to sMREs, local viscoelastic deformations govern the macroscopic relaxation response, but the different nature of the particles results in different redistribution of the particles. C) Comparison of stress relaxation tests applying external magnetic ramps of 1000 mT at different loading rates (${\dot{b}}_{\mathrm{low}}=0.2$ mT·s^−1^ and ${\dot{b}b}_{\mathrm{high}}=1000$ mT·s^−1^) on stiff (≈10 kPa) sMRE and hMRE samples. D) Detail of the responses of hMRE sample tests presented in panel (C).

While the previous results indicate that the double‐bumping effect and relaxation time modulation arise from microstructural mechanisms, we further test this hypothesis by altering the magnetic response of particles within the MRE samples. All prior experiments were conducted on sMREs, in which the magnetic particles have no residual magnetization when the external field is absent. Upon applying a magnetic field to a mechanically confined specimen, these particles magnetize, creating magnetic interactions between them and with the external field. The softness of the polymeric matrix allows for particle motion, which leads, in turn, to microstructural particle rearrangements. This fast particle mobility induces a locally heterogeneous viscoelastic loading within the matrix, resulting in an initial sharp increase in macroscopic stress. Following this, local viscous relaxation occurs, leading to an apparent reduction in stress. However, as the matrix's complex modulus (i.e., apparent stiffness) decreases during relaxation, further particle rearrangements are facilitated forming even more aligned chain‐like structures. The formation of these chains causes a secondary increase in apparent stress. During each rearrangement, new locally heterogeneous viscoelastic loading occurs followed by further viscous relaxation. These cycles of localized loading and relaxation continue until the sample reaches a magneto‐mechanical microstructural equilibrium, resulting in longer characteristic relaxation times under magnetic loading. A scheme illustrating the stages of microstructural deformation and their impact on the macroscopic mechanical response is shown in **Figure** **3**A. Notably, when the external magnetic field is applied gradually, local viscoelastic deformations are negligible, preventing the appearance of the double‐bumping effect (Figure 2C), which reveals the primordial effect of the magnetic loading rate on the observed response.

In Figure 3C, we present magnetic relaxation tests on MRE samples using the same elastomeric matrix and particle volume fraction but incorporating hard‐magnetic particles (more details on manufacturing protocol in Experimental Section). These hard‐magnetic particles retain residual magnetization even in the absence of an external magnetic field. When an external magnetic field is applied, magnetic torques are generated aligning the particles' residual magnetization with the external field. As a result, although the polymeric matrix experiences local viscoelastic loading, the microstructural response to magnetic actuation differs significantly from that of sMREs, as illustrated schematically in Figure 3B. The results in Figure 3C highlight the different responses between sMRE and hMRE samples, underscoring the microstructural nature of the effect. In addition to distinct patterns in stress evolution over time, the sMRE samples exhibit higher overall stress levels due to their larger relative magnetic permeability and saturation magnetization. A more detailed view of the macroscopic stress evolution in hMRE samples over time is provided in Figure 3D. At low magnetic application rates, an initial slight decrease in stress is observed, likely due to sample compression from local magnetic torques. Note that, to prevent loss of contact between the sample and the plate, a small pre‐compression was applied in these tests. Once the particles align their residual magnetization with the external field, they tend to form chain‐like structures that promote sample expansion, leading to a gradual increase in stress. This behavior evidences the viscous macroscopic response associated with particle alignment and chain formation. Experiments on hMRE samples have also been conducted for the soft elastomeric matrix (Figure S4, Supporting Information).

### 2.3 Viscous Driven Microstructural Rearrangement of Particles Introduces Relaxation Mechanisms in Macroscopic Sample Magnetization

**Figure 4:**
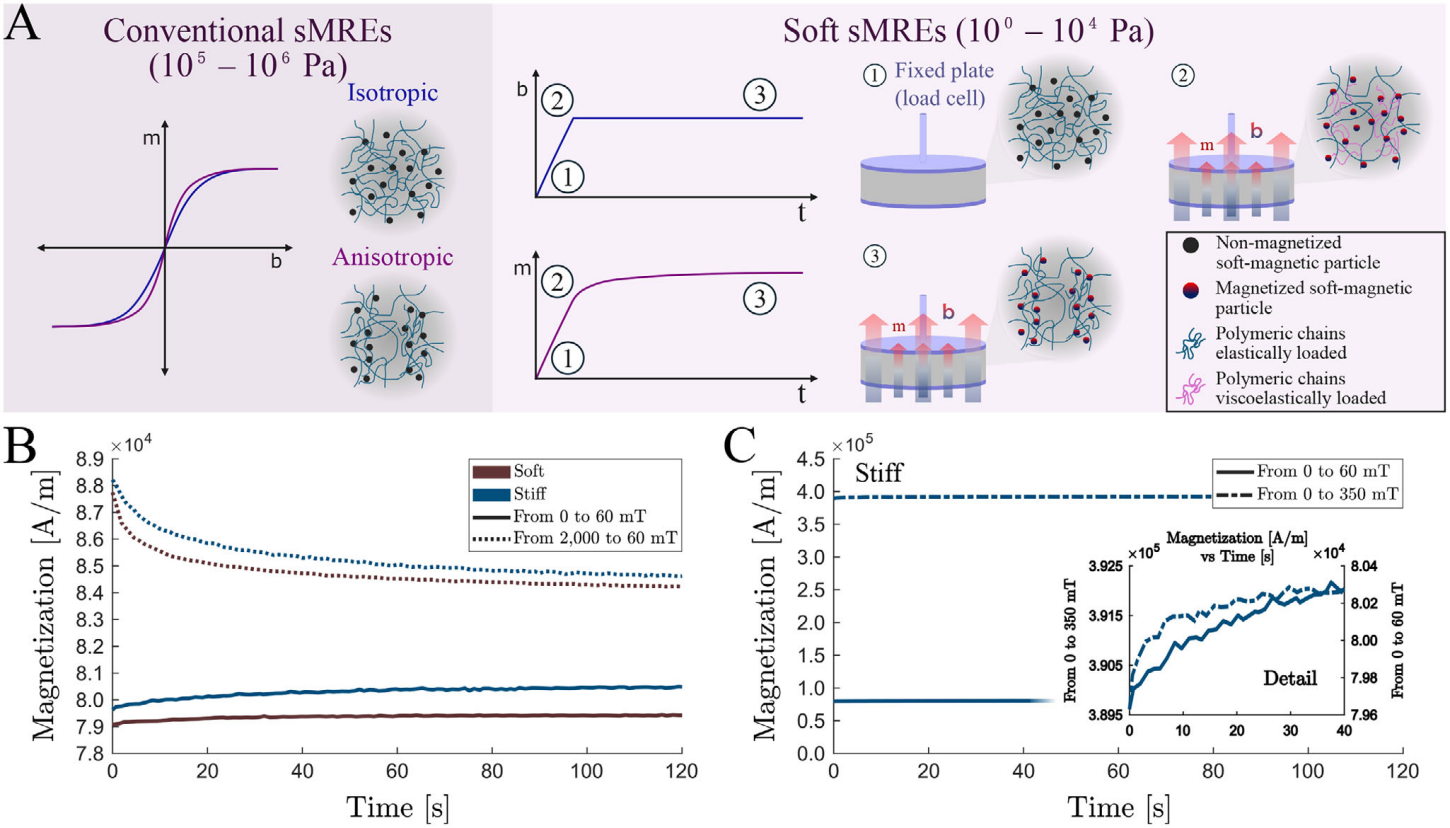
Local microstructural viscoelastic mechanisms determine macroscopic magnetization relaxation processes. A) (left) Representative magnetization versus magnetic induction curves for conventional and significantly stiffer (≈ MPa) sMREs with isotropic and anisotropic distribution of particles. Differences in magnetic permeability and magnetization at saturation are observed. (right) Schematic representation of the magnetization evolution during a fast magnetic actuation ramp in mechanically confined soft (≈ Pa to $10^{1}$ kPa) sMRE samples: 1) initial state of the sMRE; 2) formation of particle chains along the actuating direction introducing heterogeneous distributions of viscoelastic loading; 3) viscoelastic relaxation processes leading to microscopic relaxation responses in magnetization. B) Magnetization relaxation curves during magnetic actuation on soft (≈1 kPa) and stiff (≈10 kPa) sMREs. The samples are exposed to magnetic field ramp from null external magnetic field conditions, and from high magnetic fields to lower magnetic magnitudes. C) Magnetization relaxation curves, including zoom in the short‐term response, during magnetic actuation on stiff (≈10 kPa) sMREs. The samples are exposed to magnetic field ramps of different magnitudes from null external magnetic field conditions.

The findings presented in this study reveal important microstructural rearrangements occurring within soft sMREs during magnetic actuation. When a rapid external magnetic field is applied, these rearrangements lead to pronounced viscous relaxation processes within the material. Our results further illustrate how these microstructural mechanisms scale up, contributing to the nonlinear evolution of the apparent macroscopic stress. We examine next the hypothesis that this microstructural rearrangement of particles also influences the relaxation dynamics of the apparent macroscopic magnetization in the samples. This hypothesis is supported by previous experimental findings on stiffer, conventional sMRE samples with stiffness in the MPa range.^[^ ^36^, ^37^ ^]^ These prior results, schematically depicted in **Figure** **4**A, illustrate the magnetization behavior of sMREs with initially isotropic and anisotropic particle distributions. For the same particle volume fraction, both configurations exhibit similar magnetic saturation values but distinct magnetic susceptibilities. Such insights align with observations in microstructural computational models.^[^ ^13^, ^26^ ^]^ Based on the results shown in Figures 1, 2, 3 and the previous studies that compare the magnetization behavior of isotropic and anisotropic sMREs, we anticipate transient magnetization responses in our soft sMRE samples, provided the applied magnetic field is not too high for the sMRE to be in a magnetically saturated state. This transient response is likely influenced by viscoelastic processes that facilitate the formation of chain‐like particle microstructures, as illustrated in Figure 4A.

We first characterize the magnetic behavior of both soft and stiff sMRE samples. Our results indicate similar magnetic susceptibility, 1.65, and saturation magnetization, 530 kA·m^−1^ (665 mT), for both matrix stiffnesses tested (Figure S5, Supporting Information). To experimentally verify our hypothesis, we apply rapid magnetic ramps to mechanically confined samples using an MPMS3 device (Quantum Design, USA) working at 300 K. In this set of tests, rather than measuring the macroscopic stress generated, we monitor the evolution of the sample magnetic moment as a function of the applied magnetic field and time. Magnetic ramps are applied to both soft and stiff sMRE samples, reaching a final field strength of 60 mT and starting from initial field conditions of zero and 2000 mT. These findings are summarized in Figure 4C. Due to experimental constraints, we were not able to conduct long‐term tests as in the previous sections to fully capture characteristic relaxation times. Yet, the obtained results clearly indicate a transient magnetization response driven by relaxation processes. In addition, Figure 4D presents magnetization data for stiff sMRE samples at various magnetic field strengths, all below the magnetic saturation threshold. These tests support the hypothesis illustrated in Figure 4A and provide further evidence for the formation of chain‐like particle structures influenced by viscoelastic mechanisms.

### 2.4 A Theoretical Model Describes the Magneto‐Viscoelastic Microstructural Rearrangements and Motivates a Novel Actuation Mechanism Based on Mechanical Memory

Previous work in the literature suggested that magneto‐mechanical coupling can be used to introduce history dependent behavior in soft layered MRE samples through instability phenomena.^[^ ^38^ ^]^ The experimental results presented in this work reveal that, similarly but at the microstructural scale, magnetic actuation in mechanically soft sMREs induces microstructural rearrangement of particles, leading to the formation of chain‐like structures. While such microstructural formations have been previously documented in the literature, our findings underscore their significant implications for the transient coupled viscoelastic response of sMREs. Notably, we demonstrate how these formations influence key properties such as the apparent viscosity and relaxation time, as well as the emergence of a distinctive double‐bumping effect when the sample is mechanically confined. These identified microstructural mechanisms present exciting opportunities for actuation and sensing applications with integrated mechanical memory capabilities. However, the intricate interplay between mechanical and magnetic effects remains highly complex, posing significant challenges to developing clear strategies for designing and exploring solutions based on these approaches. To support this endeavor, we present a magneto‐mechanical computational framework by developing a new continuum model. This novel model captures the transient formation and rearrangement of microstructural particles under magnetic actuation, reproducing all the relevant mechanically driven viscoelastic responses reported in our experiments.

**Figure 5:**
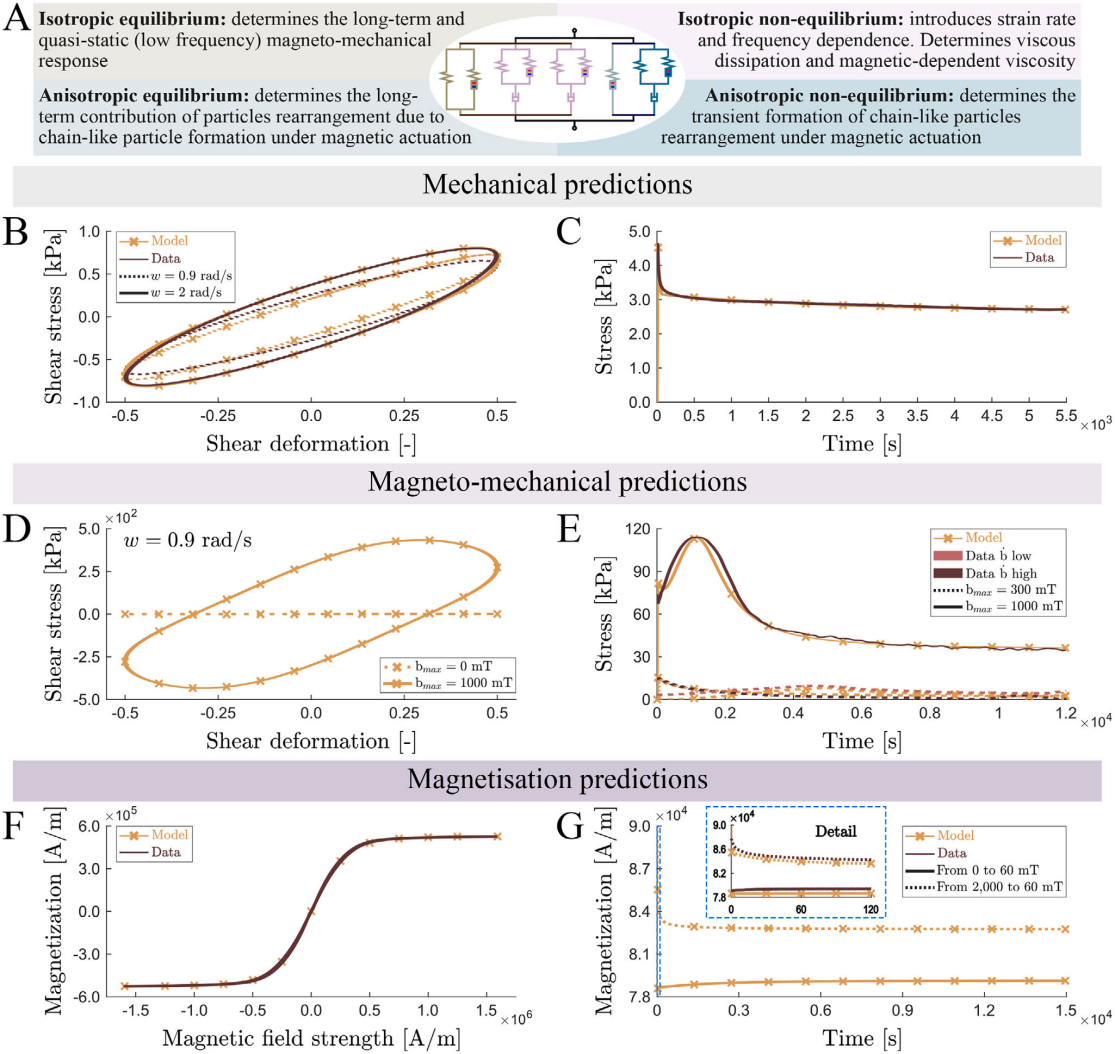
Continuum magneto‐mechanical model, motivated by microstructural arrangements, describing all coupled responses experimentally observed. A) Scheme of the rheological model that describes the finite deformation kinematics, and major theoretical considerations. B,C) Model predictions against experimental data for the purely mechanical viscous response. B) Cyclic shear loading under two different rates. C) Mechanical relaxation test. D,E) Model predictions against experimental data for magneto‐mechanical viscous response. D) Cyclic shear loading under two different rates and an external magnetic field of 1000 mT. E) Mechanically confined magnetic relaxation tests at different magnetic fields in terms of magnitude and application rate. F) Model predictions against experimental data for the magnetization response. G) Model predictions of the magnetic relaxation response (replicating experiments in Figure 4).

We propose a total energetic potential to describe the material behavior of the sMRE. An illustration of the equivalent rheological model describing the kinematics of the formulation, that consists of a combination of mechanical springs, magnetic springs, and dashpots, is presented in **Figure** **5**A. These elements are combined, forming four distinctive blocks. The first one defines an isotropic contribution that determines the magneto‐mechanical response of the material under quasi‐static loading conditions and its long‐term properties. The second block represents the isotropic contribution of viscous terms introducing strain rate and frequency dependence, which determine the viscous dissipation of the sMRE and its isotropic magnetic‐dependent viscosity. The third block defines a rate‐independent anisotropic contribution due to the formation of particle‐chain like structures under external magnetic actuation. Finally, the fourth block defines the viscous‐driven transient formation of such particle‐chains. Overall, this formulation integrates the latest magneto‐mechanical models from the literature with a novel approach that introduces the transient rearrangement of particles, driven by the interplay of magnetic and viscoelastic forces at the microstructural level. Despite the complexity of the model, the different mechanical, magnetic, and coupling mechanisms can be individually calibrated, allowing for a straightforward and rigorous relation among each branch of the constitutive model and the corresponding material responses. The details of the model formulation are provided in Experimental Section and Supporting Information.

The model predictive capabilities have been tested by comparison with experimental data in Figure 5. Figure 5B–C shows the model predictions for purely mechanical tests performed at different loading modes and strain rates. These results illustrate the capability of our formulation to describe energy dissipation and strain rate effects under shear loading (Figure 5B) and stress relaxation under uniaxial compression (Figure 5C). The magneto‐mechanical coupled response is also captured by the formulation under the different loading conditions tested. Figure 5D demonstrates the model capability to describe the viscous dependencies on magnetic actuation under shear loading, and Figure 5E its capability to describe the relaxation responses under magnetic actuation. Notably, the microstructural basis of the model formulation allows us to capture the double‐bumping effect and its dependence on magnetic field magnitude and application rate (see evolution equations in Equations (S41)– (S43), Supporting Information). Finally, in Figure 5F–G, we demonstrate the consistency of the formulation to describe the magnetization behavior of the sMRE and its transient response under fast magnetic actuation. Overall, these results establish a robust theoretical foundation for understanding the microstructural mechanisms underlying the newly reported experimental observations, while also reinforcing the conclusions outlined in the previous sections.

**Figure 6:**
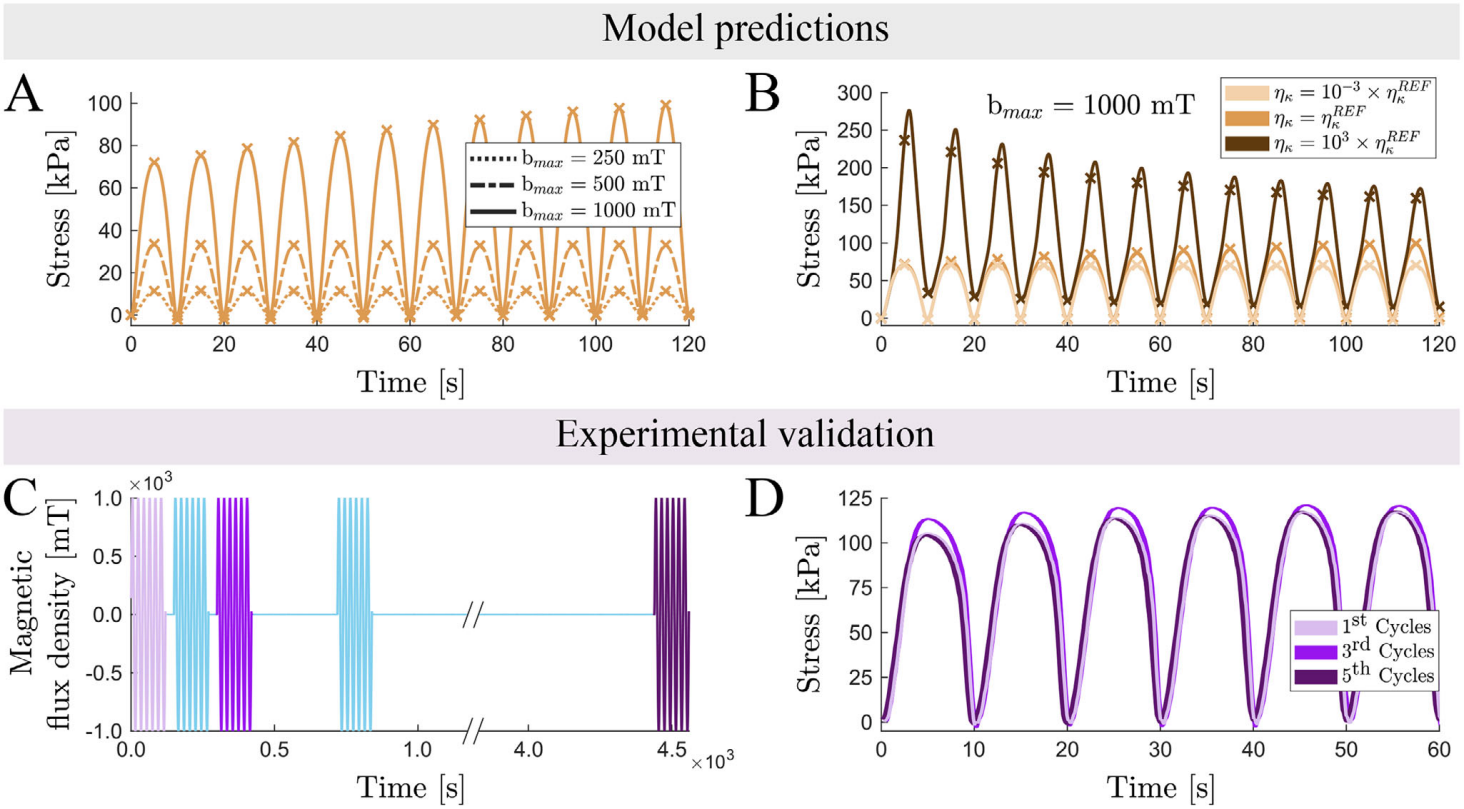
Magnetically driven viscous mechanisms can be used to design actuation responses with mechanical memory. The constitutive model developed in this work is first used to evaluate the stress response of mechanically confined sMRE samples subjected to cyclic magnetic actuation considering: A) different magnetic field magnitudes; B) different material viscosities. The history dependent stress response in these sMRE samples is then experimentally tested imposing magnetic actuation cycles with different relaxation periods between them (C). D) Stress response during the first 60 s of the first, third, and fifth magnetic cycles.

In our previous work,^[^ ^30^ ^]^ experimental tests imposing magnetic actuation on mechanically confined samples suggested the existence of a plastic regime where magnetic particle interactions induced yielding‐like responses, resulting in alternative equilibrium states. However, we now demonstrate that these observations are, in fact, manifestations of long‐term relaxation mechanisms. This insight introduces the novel concept of programming diverse magneto‐mechanical actuation behaviors through mechanical memory. The key driving factor is viscous relaxation, which is strongly modulated by magnetically driven microstructural rearrangements of the particles. With the continuum model developed in this work as a virtual testbed, we explore the material response to cyclic magnetic loading. These results highlight significant opportunities for tailoring material responses by adjusting the magnitude of the external magnetic field (**Figure** **6**A). Moreover, the thresholds for magnetic actuation can be fine‐tuned by varying the composition of (MREs), with matrix viscosity playing a pivotal role (Figure 6B). This latter dependence relies on the influence of the elastomeric viscosity and apparent stiffness to determine the characteristic formation time of particle chains.

Building on the modeling results, we aim to experimentally reproduce the observed mechanical memory effect. For this purpose, we subject mechanically confined sMRE samples to cyclic magnetic loading. To induce the mechanical memory effect, characterized by a progressive increase in stress peaks with each magnetic cycle, we apply a magnetic field with a magnitude of 1000 mT at a frequency of 0.05 Hz, as guided by the computational findings shown in Figure 6A–B. The actuation is performed using these parameters over a duration of 120 s. Three consecutive actuation sequences are conducted first, separated by a non‐actuation interval of 30 s, to analyse yielding‐like responses. Following this, a fourth actuation is applied after a 300 s interval and, finally, a fifth actuation is applied after a 1 h interval (refer to the magnetic actuation profile in Figure 6C). The corresponding experimental results are presented in Figure 6D, with additional data provided in the Figure S6 (Supporting Information). During the first actuation, a continuous increase in stress peaks is observed with successive cycles, indicative of a yielding‐like response. This behavior persists through the second and third actuations, resulting in progressively higher stress values during the initial cycles of each actuation. However, allowing the sMRE sample to relax for 1 h restores its original magneto‐mechanical response, removing the yielding‐like effects. It is important to note that the absolute stress variation is significant relative to the low modulus of such soft MREs, often exceeding the material's stiffness. In practical applications, these stress differences translate into meaningful active forces, particularly when scaled by geometry (e.g., increasing the cross‐sectional area), making them relevant for actuation or signal transmission in soft mechanical systems.

## 3 Discussion

This work has elucidated a key open question in the field of sMREs: how microstructural rearrangements during magnetic actuation influence their viscoelastic behavior. We have experimentally demonstrated that sMRE samples undergo important relaxation processes at the microstructural scale. This has been demonstrated by imposing magnetic loading on mechanically confined samples, demonstrating that they exhibit increased relaxation times by orders of magnitude compared to purely mechanical conditions. Our findings demonstrate that this modulation of the viscous response is tunable via the magnitude and rate of magnetic stimuli and, although it can be modulated by sample geometry and the nature of the magnetic filler, it is intrinsically tied to microstructural rearrangements of magnetic particles. Building on these insights, we developed magnetic actuation protocols that enable force‐memory mechanical responses in soft materials. Specifically, we induced magnetic‐driven yielding, introducing material hardening during cyclic loading, facilitated by magnetically induced long‐term viscous relaxation. This force‐memory behavior can be erased by removing the magnetic stimuli for one hour, resetting the material's performance. These mechanisms have been elucidated through a combination of advanced experimental techniques and a novel continuum model in magneto‐mechanics. In addition, the continuum model presented has proven to be an outstanding tool for uncovering new multifunctional applications based on the elucidated mechanisms.

**Figure 7:**
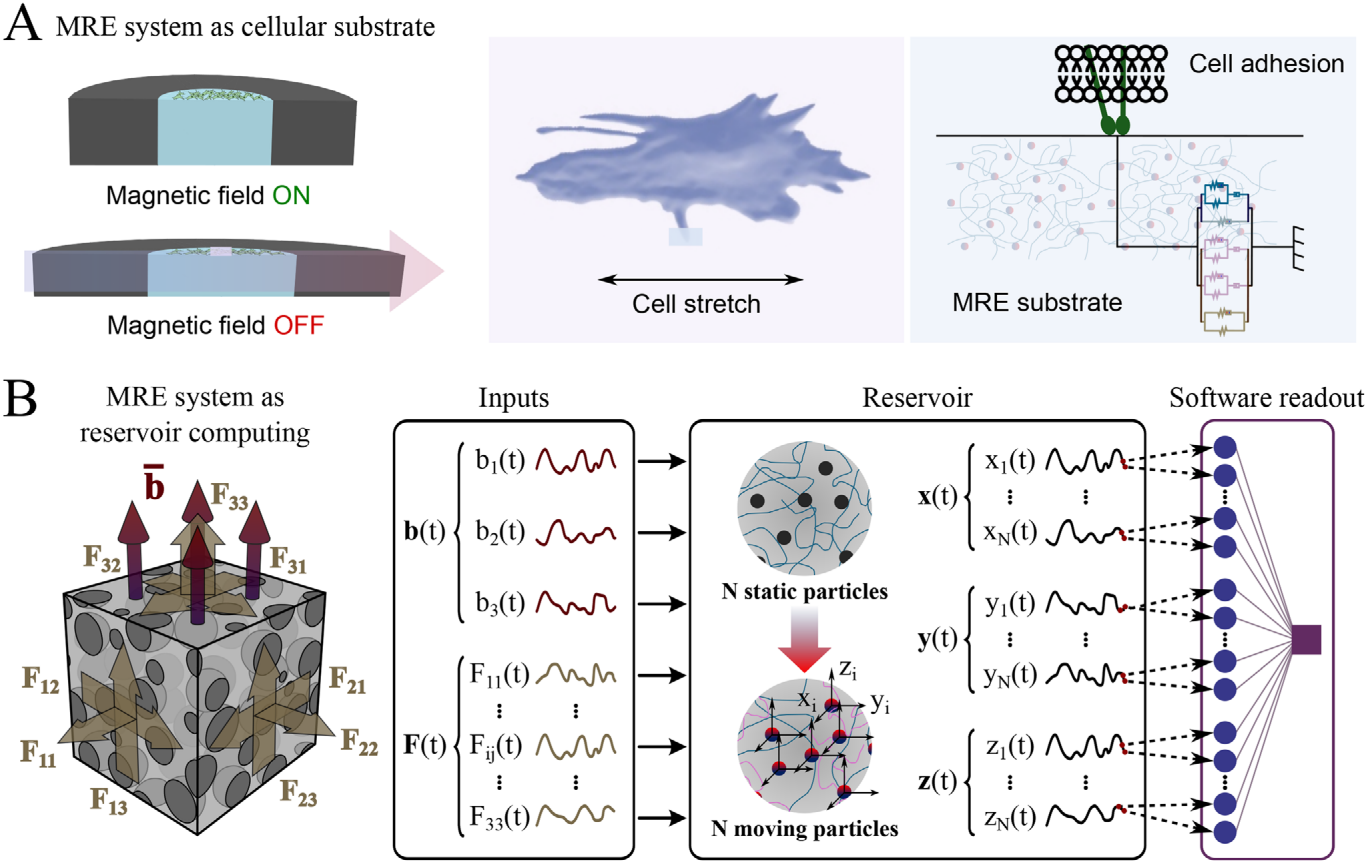
Applications for the magneto‐mechanical mechanisms identified. A) Magnetorheological elastomers (MREs) can be used as biological substrates.^[^ ^32^ ^]^ These substrates can modulate their mechanical properties and deformations in response to magnetic stimuli. These mechanical cues are transmitted to the cells, and this transmission is mediated by the viscoelastic properties of the substrate; B) MREs as reservoir computing systems. Magnetic particles act as “neurons” that, when exposed to magnetic inputs, magnetize and form a dynamic network of interactive forces transmitted through the viscous elastomer matrix. Local interactions drive particle displacement, with their motion shaped by the magnetic input's magnitude, rate, and frequency.

The results presented here address fundamental unanswered questions in the behavior of soft MREs and introduce a transformative framework for next‐generation reservoir computing systems based on nonlinear transient computation. Transient computation methods process information where time‐dependent input signals perturb the dynamics of a system. Feasible computation occurs if the resulting transient response corresponds to the input signal that triggered it. In other words, similar inputs should produce similar transients, while distinct inputs should generate distinct transients. Additionally, there must be an output mechanism capable of distinguishing between these transients and mapping them to the appropriate target output signals.^[^ ^39^ ^]^ Mechanical systems such as soft robots offer promising potential as physical reservoirs.^[^ ^40^ ^]^ Their complex body dynamics can be advantageously harnessed to produce the rich nonlinear dynamics essential for reservoir computing.^[^ ^41^ ^]^ Here, we propose mechanically confined sMRE samples as exceptional candidates for reservoir computing. To achieve this, we outline two primary approaches: 1) utilizing the sMRE sample as a macroscopic computing system, and 2) leveraging the magnetic particles as “neurons” that dynamically evolve in response to external inputs (**Figure** **7**B).

In the first approach, mechanically confined sMRE samples are subjected to time‐varying magnetic and mechanical inputs, producing a measurable macroscopic force response that evolves over time. The range of this output can be tuned through both the geometry of the sample by adjusting cross‐sectional area and exploiting edge effects; and the composition of the material, including the type and volume fraction of magnetic particles and the polymeric matrix. The magnetic input space spans three key control variables: field magnitude, application frequency, and waveform shape. Together, these parameters govern the transient and steady‐state force responses, enabling rich, nonlinear dynamics with long‐term mechanical memory, often characterized by relaxation times on the order of hours. However, the response is bounded by physical constraints, such as magnetic saturation of the particles, limitations in magnetic field homogeneity, and the intrinsic magneto‐mechanical properties of the composite. These interdependencies highlight the system's complex yet deterministic behavior, which can be further explored and optimized using in silico modeling platforms (see Figure 6). Alternatively, the nonlinear viscous behavior of the samples can be leveraged to induce complex nonlinear dynamics with short‐term mechanical memory, characterized by relaxation times ranging from seconds to minutes, through the application of mechanical inputs. In the second approach, the magnetic particles within the sMRE sample are conceptualized as “neurons”. When subjected to magnetic inputs, these particles become magnetized, generating a complex network of interactive forces that are transmitted through the viscous elastomeric matrix. The interplay of local mechanical and magnetic forces determines the relative displacements of the particles, with their temporal evolution governed by the characteristics of the magnetic inputs (e.g., magnitude, rate, and frequency). While this approach holds great promise, it faces practical limitations in experimentally tracking the particles' displacements. However, computational models that describe this microstructural coupling^[^ ^42^, ^43^, ^44^, ^45^, ^46^, ^47^, ^48^, ^49^ ^]^ can overcome these challenges, providing the necessary insights into the system's dynamics. In addition, more complex designs based on magneto‐active metastructures,^[^ ^50^, ^51^ ^]^ that resemble the microstructure of the MREs studied herein, could also be considered as potential physical reservoir systems.

Beyond the exciting prospects for reservoir computing, our findings offer valuable insights for applications in soft robotics and bioengineering. Recent studies have demonstrated the use of soft sMRE substrates as active cellular environments for mechanobiology research,^[^ ^32^ ^]^ and these approaches have been highlighted as a promising new direction in mechanomedicine.^[^ ^52^ ^]^ In such applications, the viscoelastic properties of the material, both in the short‐ and long‐term, are critical in modulating cellular behavior,^[^ ^53^ ^]^ underscoring the importance of understanding and tailoring these responses. These magnetically driven viscous responses are also crucial for magneto‐responsive soft robots that require rapid actuation or utilize viscoelastic mechanisms to trigger functional responses^[^ ^54^, ^55^ ^]^ (Figure 7A). Overall, the results presented here address previously unresolved fundamental questions in soft MREs and introduce a groundbreaking framework for developing a new generation of soft sensor‐actuator and reservoir computing systems.

## 4 Experimental Section

### Materials and Manufacturing Methods

MREs composed of a soft elastomeric matrix and soft magnetic particles were considered. The elastomeric phase used for both sMRE and hMRE samples is Dowsil CY52‐276 (DowSil, Midland, MI, USA) (PDMS), provided in two phases. The mixing ratio used for the experiments was 6:5 (“soft” samples) and 5:6 (“stiff” samples). To avoid discrepancies in the raw material, the same batch was used to manufacture all the samples. Regarding the magnetic particles, two types were used during the experiments: soft SQ carbonyl iron powder (CIP) (BASF, Germany), obtained from thermal decomposition of iron pentacarbonyl and with a mean diameter of 3.9−5 µm, and NdFeB powder (MQP‐S‐11‐9‐grade powder, Neo Materials Technology Inc., Greenwood Village, Colorado, United States), with a mean diameter of 35−55 µm. A particles' volume fraction (Φ) of 0.3 was chosen.

### Experimental Procedure for Magneto‐Mechanical Tests

A TA HR‐20 rheometer with Magneto‐Rheology Accessory from Waters TA Q600 (TA instrument, New Castle, DE, USA) was used. This Magneto‐Rheology Accessory creates a magnetic field in the axial direction to the cylindrical samples. Contrary to other devices where the magnetic field strength was set to a certain value, this device used a closed loop system to control the magnetic flux density by measuring it in the vicinity of the sample. It was also capable of measuring and controlling the temperature thanks to the integrated cooling system.

This device was used to perform: i) oscillatory shear tests and ii) mechanically confined tests under different magnetic field functions. The first tests were conducted with constant amplitude and frequency on cylindrical sMRE samples. The device was programmed to apply a torque on the cylindrical sample achieving a sine‐shaped angular displacement. This deformation profile was defined for a 50% strain and fixed angular velocities of w={0.1,0.9,2.0} rad·s^−1^.^[^ ^30^ ^]^ Furthermore, these tests were carried out under different magnetic flux densities of B={0,200,500,1000} mT. The mechanically confined tests were performed by applying a magnetic field function coaxial to the cylindrical sample while mechanically confining the composite in the axial direction. Two functions were used: sinusoidal magnetic field function with different frequencies (f={0.02,0.05,0.10} Hz), and magnetic ramp functions characterized by an initial linear slope ($\dot{B}=\left\{300,1000\right\}$ mT·s^−1^) and a constant target magnetic flux density (B={300,700,1000} mT). Once a specific targeted magnetic field was reached, it was kept constant during certain time (to reach steady state, i.e., for about 12000 s).

### Experimental Procedure for Magnetization Tests

To analyze the magnetization behavior, the composite was cut with a cylindrical shape and immobilized into a plastic cylinder of the same size (VSM Powder sample holders QDC‐4096‐388, Quantum Design, USA). Then, after accurately weighting, the immobilized composite was placed in a brass half‐tube sample holder (Quantum Design, USA). Measurements were performed in a MPMS3 device (Quantum Design, USA) at a temperature of 300 K, collecting the sample magnetic moment as a function of the applied magnetic field and time.

Regarding the application of a magnetic field, two different approaches were used. First, the whole hysteresis curve was recorded in field dependent magnetization measurements between 2 and −2 T at 300 K. In addition, a constant magnetic field (either 60, 350, and 2000 mT) was applied and the magnetization of the sample was recorded over time. The last type of measurements was performed in two different ways. The first option was by stabilizing the magnetic field at 0 T, then applying the target field (at 500 Oe·s^−1^) and finally measuring the magnetization evolution over the following 2 min. The second option consisted in applying a saturation magnetization field (2 T), then reducing the magnetic field to the target field (at 50 mT·s^−1^) and finally recording the magnetization variation over time.

### Continuum Framework for Magneto‐Mechanical Materials with Viscous‐Driven Particle Alignment

A continuum framework was developed to capture the dynamic particle alignment due to deep magnetic and mechanical coupled processes. The main hypotheses and constitutive definitions were provided here. The full description of the model is described further in Supporting Information.

### Kinematics and Magnetic Variables

Given the large deformation capability of these materials, the framework followed a finite strain theory formalism. In order to include time‐dependent responses in the framework, the deformation grandient F was decomposed into elastic ($F^{e}$) and viscous ($F^{v}$) contributions,

(1) $$F=F^{e}F^{v}.$$

The magnetic variables of relevance were the nominal magnetic field vector and the nominal magnetic flux density vector, which could be respectively expressed in the material (reference) form as H and B, and in the spatial (current) configuration as h and b. These variables can be related via the transformations

(2) $$h=F^{-T}H$$

and

(3) $$b=J^{-1}FB$$

The magnetization vector, m, expressed in the current configuration, arises when adopting the following constitutive equation^[^ ^56^ ^]^ relating the three of them:

(4) $$b=\mu_{0}\left(h+m\right)$$

where $\mu_{0}$ is the magnetic permeability constant of free space with a value of $4\pi \times 10^{-7}$ H·m^−1^. Note that there was non‐uniqueness of the Lagrangian form of m. Some recent works point out that, in incompressible isotropic MREs, m should not depend on the stretch U but on the body rotation R,^[^ ^46^, ^57^, ^58^ ^]^ resulting in

(5) $$m=RM\;\text{with}\;R=FU^{-1},$$

where M denotes some reference magnetization measure. In the present setting, the magnetization was not used directly as an independent variable but was only post‐processed by knowledge of H and B.

### Governing Equations

The coupled problem was defined by the linear momentum and angular momentum principles, and the uncoupled Maxwell's equations. The linear momentum balance, neglecting inertial terms, can be expressed in the material configuration as

(6) $$\nabla_{0}\cdot P_{\text{tot}}+B_{f}=0\;\;\;\;\;\text{on}\;\;\;\;\;\Omega_{0}$$

(7) $$-[[P_{\text{tot}}]]\cdot N^{+}=T_{\text{tot}}\;\;\;\;\;\text{on}\;\;\;\;\;\partial \Omega_{0}$$

where $P_{\text{tot}}$ is the total first Piola stress tensor, $B_{f}$ denotes the external mechanical body force vector, $[[\;\cdot \;]]:={\left[\;\cdot \;\right]}^{+}-{\left[\;\cdot \;\right]}^{-}$ is defined as the jump in a quantity across the boundary, $N^{+}$ is the outward unit normal to the nominal boundary surface and $T_{\text{tot}}$ refers to the nominal traction on the surface of the body, $\partial \Omega_{0}$.

In relation to the angular momentum principle, a tensor symmetry condition results from it

(8) $$P_{\text{tot}}F^{T}=FP_{\text{tot}}^{T}.$$

Under magnetostatic assumptions (no currents and charges), the magnetic part of the Maxwell's equations can be reduced to

(9) $$\nabla_{0}\times H=0,\;\nabla_{0}\cdot B=0\;\;\;\;\;\text{on}\;\;\;\;\;\Omega_{0}\cup S_{0}$$

(10) $$N^{+}\times [[H]]=0,\;N^{+}\cdot [[B]]=0\;\;\;\;\;\text{on}\;\;\;\;\;\partial \Omega_{0}$$

where $S_{0}$ represents the magnetically permeable free space in which the deformable body is immersed. Furthermore, by assuming that H derives from a scalar potential field φ, i.e.,

(11) $$H=-\nabla_{0}\varphi ,$$

the Ampère–Maxwell law, Equation (9)_1_, is automatically satisfied.

### Thermodynamics

Considering isothermal conditions, the total strain energy potential per unit reference volume, Ψ, of a magneto‐mechanical problem comprising MREs could be defined as a function of the deformation gradient, the nominal magnetic field vector^[^ ^56^, ^59^ ^]^ and a set of scalar, vectorial and tensorial internal variables. Among the latter, mechanical viscous dissipation was considered via viscous contributions to the deformation gradient ($F_{i}^{v}$). Given the dynamic and highly non‐linear nature of the particles rearrangement process, leading to a rapid change from a completely isotropic material to a transversely isotropic one, two new additional internal variables were introduced. The first variable, $n_{A}$, was a unit vector which points in the direction of the chain formation. The second internal variable, $\kappa_{A}$, is a scalar variable representing the chain dispersion in fractional anisotropy theory, i.e., it described the degree of material anisotropy.^[^ ^60^ ^]^

The total Helmholtz free energy potential is expressed as

(12) $${\hat{\Psi }}_{\text{total}}\left(F,H,F_{i}^{v},\kappa_{A},n_{A}\right)=\hat{\Psi }\left(F,H,F_{i}^{v},\kappa_{A},n_{A}\right)-\frac{\mu_{0}}{2}J\left[H⊗H\right]:C^{-1}\left(F,H\right)$$

In this last equation, $C=F^{T}F$ denotes the right Cauchy‐Green deformation tensor, whereby the last term serves to describe the energy stored in the magnetic free space corresponding to $\Omega_{0}\cup S_{0}$. More generally, in order to automatically satisfy the objectivity theorem,^[^ ^61^ ^]^ Ψ can be defined in terms of the invariants $\left\{I_{1},\text{…},I_{10}\right\}$ of **C** and H (see definitions in Supporting Information).

From the development of thermodynamics principles and through the application of the Coleman‐Gurtin^[^ ^62^ ^]^ or Coleman‐Noll^[^ ^63^ ^]^ procedures, the following constitutive relations are obtained

(13) $$P=\frac{\partial \Psi_{\text{total}}}{\partial F}-pF^{-T}$$

(14) $$B=-\frac{\partial \Psi_{\text{total}}}{\partial H}.$$

### Energy Functions and Constitutive Equations

The strain energy per unit reference volume (without the free space term, Equation (12)), $\Psi (F,H,F_{i}^{v},\kappa_{A},n_{A})$, was defined as a sum of magnetization, $\Psi_{\text{mag}}\left(F,H,\kappa_{A},n_{A}\right)$, isotropic, $\Psi_{\text{iso}}\left(F,H,F_{i}^{v}\right)$, and anisotropic contributions, $\Psi_{\text{ani}}\left(F,H,F_{i}^{v},\kappa_{A},n_{A}\right)$. Furthermore, the last two contributions could be further decomposed into equilibrium and non‐equilibrium terms as

(15) $$\begin{array}{cc} & \hat{\Psi }\left(F,H,F_{i}^{v},\kappa_{A},n_{A}\right)={\hat{\Psi }}_{\text{mag}}\left(F,H,\kappa_{A},n_{A}\right)+{\hat{\Psi }}_{\text{iso}}^{\text{eq}}\left(F,H\right)+{\hat{\Psi }}_{\text{iso}}^{\text{neq,st}}\left(F,H,F_{\text{iso,st}}^{v}\right)\\ & +{\hat{\Psi }}_{\text{iso}}^{\text{neq,lt}}\left(F,H,F_{\text{iso,lt}}^{v}\right)+{\hat{\Psi }}_{\text{ani}}^{\text{eq}}\left(F,H,\kappa_{A},n_{A}\right)+{\hat{\Psi }}_{\text{ani}}^{\text{neq}}\left(F,H,F_{\text{ani}}^{v},\kappa_{A},n_{A}\right) \end{array}$$

It should be noted that, since the behavior of this family of MREs was strongly dependent on the magnetic loading, the magnetic contributions woud be included inside each of the terms of the expression above. A rheological representation of the model can be seen in Figure 5.

The magnetization term is defined as
(16)

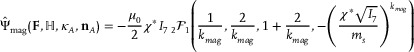

where

(17) $$\begin{array}{c} \chi^{*}=\chi \left[1+q_{\mathrm{mag}}\text{tanh}\left(p_{\mathrm{mag}}I_{9}\right)\right] \end{array};$$

χ and $m_{s}$ are the magnetic susceptibility and the magnetic saturation, respectively; 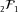 denotes de hypergeometric function; $k_{\mathrm{mag}}$ is an exponent controlling how fast the material saturates; tanh refers to the hyperbolic tangent function and $q_{\mathrm{mag}}$ and $p_{\mathrm{mag}}$ are material parameters that estimate the impact of the particles chains formation on the magnetization. Note that the last term in Equation (17) introduces a direct dependence of the material magnetization upon the particle chain formation and evolution with applied magnetic loading (as shown in experiments).

The equilibrium part of the isotropic term was defined by a Neo‐Hookean energy potential, with $\mu_{iso}^{eq}$ being the shear modulus

(18) $${\hat{\Psi }}_{\text{iso}}^{\text{eq}}\left(F,H\right)=\frac{\mu_{iso}^{eq}}{2}\left(I_{1}-3\right)$$

The non‐equilibrium part was split into two principal branches that represent short‐term and long‐term relaxation mechanisms. The short‐term potential is defined as

(19) $$\begin{array}{cc} {\hat{\Psi }}_{\text{iso}}^{\text{neq,st}}\left(F,H,F_{\text{iso,st}}^{v}\right) & =\frac{\mu_{iso}^{neq,st}}{2}\left(1+\frac{q_{0}I_{7}^{e,iso,st}}{1+\frac{q_{0}}{q_{s}}I_{7}^{e,iso,st}}\right)\left(I_{1}^{e,iso,st}-3\right) \end{array}$$

with $I_{1}^{e,iso,st}=\text{tr}\left({\left(F_{\text{iso,st}}^{v}\right)}^{-T}F^{T}F{\left(F_{\text{iso,st}}^{v}\right)}^{-1}\right)=\text{tr}\left(C_{\text{iso}}^{e}\right)$. In the expression above, $\mu_{iso}^{neq,st}$ refers to the shear modulus of the short‐term non‐equilibrium part; $q_{0}>0$ describes the initial response of the magnetic coupling and $q_{s}>0$ sets an upper saturation value to it.^[^ ^64^ ^]^ On the other hand, the long‐term non‐equilibrium part of the isotropic contribution is defined as

(20) $$\begin{array}{cc} {\hat{\Psi }}_{\text{iso}}^{\text{neq,lt}}\left(F,H,F_{\text{iso,lt}}^{v}\right) & =\frac{\mu_{iso}^{neq,lt}}{2}\left(I_{1}^{e,iso,lt}-3\right)\\ & +\left[\left(1+\theta^{neq}\right)f\left(I_{4}^{e,iso,lt}\right)+\left(1-\theta^{neq}\right)f\left(I_{8}^{e,iso,lt}\right)-2f\left(I_{7}^{e,iso,lt}\right)\right] \end{array}$$

where $\mu_{iso}^{neq,lt}$ is the shear modulus of the long‐term non‐equilibrium part; $\theta^{\mathrm{neq}}$ is a constant, and $f\left(I_{i}^{j}\right)$ has the following expression

(21) $$\begin{array}{c} f\left(I_{i}^{j}\right)=\frac{\beta_{1}^{j}\mu_{0}m_{s}^{2}}{2\beta_{2}^{j}\chi }\log\left(1+\frac{\beta_{2}^{j}\chi^{2}}{m_{s}^{2}}I_{i}\right)\;\text{with}\;j\in \left\{\mathrm{eq},\mathrm{neq}\right\} \end{array},$$

where $\beta_{1}^{j}$ and $\beta_{2}^{j}$ are constants.

The last two terms of the strain energy potential, $\Psi_{\text{ani}}^{\text{eq}}$ and $\Psi_{\text{ani}}^{\text{neq}}$, were also based on the Neo‐Hookean model using $I_{10}$ instead of $I_{1}$. Moreover, $I_{9}$ was subtracted from $I_{10}$ to constrain the material to isochoric behavior. Letting next $\Psi_{\text{ani}}^{\text{eq}}$ comprise coupled magneto‐mechanical mechanisms, those last two terms are defined as

(22) $$\begin{array}{cc} {\hat{\Psi }}_{\text{ani}}^{\text{eq}}\left(F,H,\kappa_{A},n_{A}\right) & =\text{tanh}\left(w_{0}I_{9}\right)\left[\left(1+\theta^{eq}\right)f\left(I_{4}\right)+\left(1-\theta^{eq}\right)f\left(I_{8}\right)-2f\left(I_{7}\right)\right] \end{array}$$

(23) $$\begin{array}{cc} {\hat{\Psi }}_{\text{ani}}^{\text{neq}}\left(F,H,F_{\text{ani}}^{v},\kappa_{A},n_{A}\right) & =\frac{\mu_{ani}^{neq}}{2}\left(I_{1}^{e,ani}-3\right)+\frac{\alpha_{ani}\mu_{ani}^{neq}}{2}\left(I_{10}^{e,ani}-I_{9}\right) \end{array}$$

Here, $\mu_{\mathrm{ani}}^{\mathrm{neq}}$ is a material parameter with the same meaning as in the standard Neo‐Hookean model; tanh refers to the hyperbolic tangent function; $w_{0}$ and $\theta^{\mathrm{eq}}$ are constants, $\alpha_{\mathrm{ani}}$ is a scaling factor and $f(I_{i}^{j})$ is defined in Equation (21).Finally, we need to define the evolution equations for the internal variables $F_{\text{iso,st}}^{v}$, $F_{\text{iso,lt}}^{v}$, $F_{\text{ani}}^{v}$, $\kappa_{A}$ and $n_{A}$. These definitions are provided and discussed in Supporting Information.

## Conflict of Interest

The authors declare no conflict of interest.

## Author Contributions

All authors conceptualized the study. E.G.S., M.L.L.D., and D.G.G. conducted the magneto‐mechanical experiments. L.G. conducted the magnetic experiments. E.G.S, K.D and D.G.G developed the constitutive model. E.G.S and D.G.G wrote the original manuscript. All authors conducted the formal analysis and discussion and revised the paper.

## Supporting information

Supporting Information

## Data Availability

The data that support the findings of this study are available from the corresponding author upon reasonable request.
