# Supplementary material for: Magnetic‐Driven Viscous Mechanisms in Ultra‐Soft Magnetorheological Elastomers Offer History‐Dependent Actuation with Reprogrammability Options
Source: Adv Sci (Weinh). 2025 Aug 13;12(35):e06790. doi: 10.1002/advs.202506790 (PMC12463005; doi:10.1002/advs.202506790)
Supplement: Supplementary file 1 — Supporting Information [file ADVS-12-e06790-s001.pdf]

ADVANCED SCIENCE
Open Access

Supporting Information

for *Adv. Sci.*, DOI 10.1002/advs.202506790

Magnetic-Driven Viscous Mechanisms in Ultra-Soft Magnetorheological Elastomers Offer History-Dependent Actuation with Reprogrammability Options

*Ernesto Gonzalez-Saiz, Maria Luisa Lopez-Donaire, Lucía Gutiérrez, Kostas Danas and Daniel Garcia-Gonzalez**

SUPPORTING INFORMATION
Magnetic-driven viscous mechanisms in ultra-soft magnetorheological elastomers offer history-dependent actuation with reprogrammability options

*Ernesto Gonzalez-Saiz[1] Maria Luisa Lopez-Donaire[1] Lucía Gutiérrez[2] Kostas Danas[3] Daniel Garcia-Gonzalez[1]**

E. Gonzalez-Saiz, M. L. Lopez-Donaire, D. Garcia-Gonzalez
[1]Department of Continuum Mechanics and Structural Analysis, Universidad Carlos III de Madrid, Calle Butarque 15, Leganes, 28911, Madrid, Spain
Email Address: danigarc@ing.uc3m.es

L. Gutiérrez
[2]Instituto de Nanociencia y Materiales de Aragón (INMA, CSIC/UNIZAR) and CIBER-BBN, 50018, Zaragoza, Spain

K. Danas
[3]LMS, CNRS, École Polytechnique, Institut Polytechnique de Paris, Palaiseau, 91128, France

1 Continuum framework for magneto-mechanical materials with viscous-driven particle alignment

In this section, we present the continuum framework developed to capture the dynamic particle alignment due to strongly coupled magnetic and mechanical processes. First, we present the kinematics of the model together with the magnetic variable definitions. Then, we formulate the governing equations and, finally, derive constitutive relations by use of non-equilibrium thermodynamic considerations.

1.1 Kinematics and magnetic variables

The magnetorheological elastomers (MREs) used in this work are made up of a soft polymeric matrix and isotropically distributed magnetic particles. The overall response of this material results from a combination of purely mechanical deformation contributions and magnetically-induced ones.

Given the large deformation capability of the present materials, the proposed framework follows a finite strain theory formulation. To this end, a nonlinear deformation map, $\boldsymbol{\psi}$, relates the material (undeformed) configuration $\boldsymbol{\Omega_0}$, where points are identified with the material (reference or undeformed) coordinates $\mathbf{X}$, to a spatial (current or deformed) configuration $\boldsymbol{\Omega}$, where the same points are projected to the spatial coordinates $\mathbf{x}$. Using this mapping process, one may define the deformation gradient $\mathbf{F}$ with components in a Cartesian laboratory frame, $F_{ij} = \nabla_{0,j}\psi_i = \partial x_i / \partial X_j$. Moreover, the change in volume may be defined in terms of the Jacobian $J = \det(\mathbf{F}) > 0$. Note that ∇_0 refers to the gradient operator with respect to the material coordinates $\mathbf{X}$. Furthermore, we introduce the right Cauchy-Green tensor, $\mathbf{C} = \mathbf{F}^{\mathrm{T}}\mathbf{F}$, which is useful to define the subsequent invariants since it is an objective tensor of deformation measure.

We introduce the viscous rate effects in the framework by considering a multiplicative decomposition of $\mathbf{F}$ [1, 2] into elastic ($\mathbf{F}^e$) and viscous ($\mathbf{F}^v$) contributions, i.e.,

$$\mathbf{F} = \mathbf{F}^e\mathbf{F}^v. \tag{1}$$

This allows for the definition of a non-equilibrium right Cauchy-Green deformation tensor as

$$\mathbf{C}^e = (\mathbf{F}^e)^{\mathrm{T}}\mathbf{F}^e. \tag{2}$$

On the other hand, the magnetic variables of relevance are the magnetic field vector and the magnetic flux density vector, which can be respectively expressed in material form as $\mathbb{H}$ and $\mathbb{B}$, and in the spatial configuration as h and b. These variables are related by

$$h = \mathbf{F}^{-\mathrm{T}}\mathbb{H}, \tag{3}$$

$$b = J^{-1}\mathbf{F}\mathbb{B}. \tag{4}$$

In addition to the aforementioned magnetic variables, the magnetization vector, m, expressed in the current configuration, arises when adopting the following constitutive equation [3] relating the three of them:

$$b = \mu_0(h + m), \tag{5}$$

where μ_0 is the magnetic permeability constant of free space with a value of $4\pi \times 10^{-7}$ H·m^{-1}. Note that there is non-uniqueness of the Lagrangian form of m. Recent works point out that, in

the context of incompressible MREs (as is the present case), m does not depend on the stretch $\mathbf{U}$ but on only on the rotation $\mathbf{R}$ [4, 5, 6, 7], resulting in

$$m = \mathbf{R}\mathbb{M} \quad \text{with} \quad \mathbf{R} = \mathbf{F}\mathbf{U}^{-1}. \tag{6}$$

1.2 Governing equations

As in any magneto-mechanics problem regarding MREs, the relevant governing equations derive from the linear momentum and angular momentum principles, and the uncoupled Maxwell's equations. The linear momentum balance, neglecting inertial terms, can be expressed in the material configuration as

$$\nabla_0 \cdot \mathbf{P}_{\text{tot}} + \mathbf{B}_{\text{f}} = \mathbf{0} \quad \text{on} \quad \Omega_0 \tag{7}$$

$$-[\![\mathbf{P}_{\text{tot}}]\!] \cdot \mathbf{N}^+ = \mathbf{T}_{\text{tot}} \quad \text{on} \quad \partial\Omega_0 \tag{8}$$

where $\mathbf{P}_{\text{tot}}$ is the total first Piola stress tensor, $\mathbf{B}_{\text{f}}$ denotes the external mechanical body force vector, $[\![\cdot]\!] := [\cdot]^+ - [\cdot]^-$ is defined as the jump in a quantity across the boundary, $\mathbf{N}^+$ is the outward unit normal to the nominal boundary surface and $\mathbf{T}_{\text{tot}}$ refers to the nominal traction on the surface of the body, $\partial\Omega_0$.
Use of conservation of angular momentum leads to the symmetry condition

$$\mathbf{P}_{\text{tot}}\mathbf{F}^{\text{T}} = \mathbf{F}\mathbf{P}_{\text{tot}}^{\text{T}} \quad \text{or} \quad \boldsymbol{\sigma}_{\text{tot}} = \boldsymbol{\sigma}_{\text{tot}}^{\text{T}}. \tag{9}$$

The total first Piola stress tensor and the total Cauchy stress tensor $\boldsymbol{\sigma}_{\text{tot}}$ are related by

$$\boldsymbol{\sigma}_{\text{tot}} = J^{-1}\mathbf{P}_{\text{tot}}\mathbf{F}^{\text{T}}. \tag{10}$$

Finally, under magnetostatic assumptions, i.e., very slow electro-magnetic fields changes and absence of free electric currents in the MRE or the particles, the Maxwell's equations can be reduced to

$$\nabla_0 \times \mathbb{H} = \mathbf{0}, \quad \nabla_0 \cdot \mathbb{B} = 0 \quad \text{on} \quad \Omega_0 \cup \mathcal{S}_0 \tag{11}$$

$$\mathbf{N}^+ \times [\![\mathbb{H}]\!] = \mathbf{0}, \quad \mathbf{N}^+ \cdot [\![\mathbb{B}]\!] = 0 \quad \text{on} \quad \partial\Omega_0 \tag{12}$$

where $\mathcal{S}_0$ represents the magnetically permeable free space in which the deformable body is immersed. Furthermore, by assuming that $\mathbb{H}$ derives from a scalar potential field φ, the Ampère–Maxwell law, Eq. $(11)_1$, is automatically satisfied by definition with

$$\mathbb{H} = -\nabla_0\varphi. \tag{13}$$

1.3 Maxwell stress

The influence of the magnetic field on the mechanical stress in the deforming body is incorporated through a magnetic stress tensor, being $\mathbf{T}_{\text{tot}}$ in Eq. (8) the combination of the mechanical tractions from boundary conditions and those associated with the Maxwell stress. The Maxwell stress is defined as

$$\mathbf{P}_{\text{maxw}} = \left[-\frac{\mu_0}{2}\left[\mathbb{H} \otimes \mathbb{H}\right] : J\mathbf{C}^{-1}\right]\mathbf{F}^{-\text{T}} + \left[\mathbf{F}^{-\text{T}}\mathbb{H}\right] \otimes \mathbb{B}. \tag{14}$$

In vacuum (i.e., no magnetization), the Maxwell stress reduces to

$$\mathbf{P}_{\text{maxw}} = \left[-\frac{\mu_0}{2}\left[\mathbb{H} \otimes \mathbb{H}\right] : J\mathbf{C}^{-1}\right]\mathbf{F}^{-\text{T}} + \mu_0 J\left[\mathbf{F}^{-\text{T}}\mathbb{H}\right] \otimes \left[\mathbf{C}^{-1}\mathbb{H}\right]. \tag{15}$$

1.4 Thermodynamics

Assuming isothermal conditions, the total strain energy potential per unit reference volume, Ψ, of a magneto-mechanical problem comprising MREs may be defined as a function of the deformation gradient, the magnetic field vector [3, 8] and a set of scalar, vectorial and tensorial internal variables. Among the latter, mechanical viscous dissipation is considered via viscous contributions to the deformation gradient ($\mathbf{F}_i^v$). Given the time-evolving nature of the particles rearrangement, changing from a completely isotropic material to a transversely isotropic one, two new additional internal variables are introduced. The first variable, $\mathbf{n}_A$, is a unit vector which points in the direction of the chain formation. The second internal variable, κ_A, is a scalar variable representing the chain dispersion in fractional anisotropy theory, i.e., it describes the degree of material anisotropy.
The total Helmholtz free energy potential is expressed as

$$\hat{\Psi}_{\text{total}}(\mathbf{F}, \mathbb{H}, \mathbf{F}_i^v, \kappa_A, \mathbf{n}_A) = \hat{\Psi}(\mathbf{F}, \mathbb{H}, \mathbf{F}_i^v, \kappa_A, \mathbf{n}_A) - \frac{\mu_0}{2} J \left[\mathbb{H} \otimes \mathbb{H}\right] : \mathbf{C}^{-1}, \tag{16}$$

where the last term in the above equation is the energy stored in the magnetic free space corresponding to $\Omega_0 \cup \mathcal{S}_0$. To automatically satisfy the objectivity theorem [9], Ψ can be defined in terms of the invariants of $\mathbf{C}$. The first three invariants, related to an isotropic medium, are defined as

$$I_1(\mathbf{C}) = \text{tr}(\mathbf{C}), \tag{17}$$

$$I_2(\mathbf{C}) = \frac{1}{2}[(\text{tr}(\mathbf{C}))^2 - \text{tr}(\mathbf{C}^2)], \tag{18}$$

$$I_3(\mathbf{C}) = \det(\mathbf{C}) = J^2. \tag{19}$$

Some additional irreducible invariants must be included to satisfy the representation theorem [10] for a magneto-elastic problem [11]. They consist of a purely magnetostatic and four coupled magneto-elastic ones, which read as

$$I_4(\mathbb{H}) = [\mathbb{H} \otimes \mathbb{H}] : \mathbf{I}, \tag{20}$$

$$I_5(\mathbf{C}, \mathbb{H}) = [\mathbb{H} \otimes \mathbb{H}] : \mathbf{C}, \quad I_6(\mathbf{C}, \mathbb{H}) = [\mathbb{H} \otimes \mathbb{H}] : \mathbf{C}^2, \tag{21}$$

$$I_7(\mathbf{C}, \mathbb{H}) = [\mathbb{H} \otimes \mathbb{H}] : \mathbf{C}^{-1} = \frac{1}{I_3}\left[I_6 - I_1 I_5 + I_2 I_4\right], \tag{22}$$

$$I_8(\mathbf{C}, \mathbb{H}) = [\mathbb{H} \otimes \mathbb{H}] : \mathbf{C}^{-2}, \tag{23}$$

where $\mathbf{I}$ denotes the second-order identity tensor and the symbols $:$ and $\otimes$ represent the double contraction and the tensor product, respectively. Note that I_7 can be written in terms of the other invariants by following the Cayley-Hamilton theorem [12].
To introduce the dynamic microstructural rearrangements identified from the experiments, we include two state variables that describe the formation of particle-chain like structures considering their main direction ($\mathbf{n}_A$) and the degree of anisotropic distribution (κ_A). Based on these variables, two additional invariants are defined as

$$I_9(\mathbf{C}, \kappa_A, \mathbf{n}_A) = \text{tr}\left(\mathbf{G}^*\right) \quad \text{and} \quad I_{10}(\mathbf{C}, \kappa_A, \mathbf{n}_A) = \mathbf{C}^{-1} : \mathbf{G}^*. \tag{24}$$

Note that the term $\mathbf{G}^* = [1 - 3\kappa_A]\,\mathbf{n}_A \otimes \mathbf{n}_A$ is a modification of the standard structural tensor in fractional anisotropy, usually taking the form $\mathbf{G} = \kappa\mathbf{I} + [1 - 3\kappa]\,\mathbf{n} \otimes \mathbf{n}$. Following that fractional anisotropy theory, $\kappa_A \in [0, 1/3]$ with $\kappa_A = 0$ representing a perfect transversely isotropic material and $\kappa_A = 1/3$ a purely isotropic one. Since this work mainly focuses on the formation of magnetically derived microstructural rearrangements, we have decided to split the isotropic and anisotropic contributions into different invariants, with I_9 and I_{10} being purely anisotropic. Due to the experimental observations showing a strong influence of viscous mechanisms on the magneto-mechanical behavior of these materials, equivalent invariants based on $\mathbf{C}^e$ are also considered.

The second law of thermodynamics is expressed in the form of the Clausius-Duhem dissipation inequality as [13, 14]

$$\begin{aligned}
\mathcal{D}_{\text{int}} &= \mathbf{P} : \dot{\mathbf{F}} + p\mathbf{F}^{-\mathrm{T}} : \dot{\mathbf{F}} - \mathbb{B} \cdot \dot{\mathbb{H}} - \dot{\Psi}_{\text{total}}(\mathbf{F}, \mathbb{H}, \mathbf{F}_i^v, \kappa_A, \mathbf{n}_A) - \theta\,(\Gamma_0^{\kappa_A} + \Gamma_0^{\mathbf{n}_A}) = \\
&= \mathbf{P} : \dot{\mathbf{F}} + p\mathbf{F}^{-\mathrm{T}} : \dot{\mathbf{F}} - \mathbb{B} \cdot \dot{\mathbb{H}} - \frac{\partial \Psi_{\text{total}}}{\partial \mathbf{F}} : \dot{\mathbf{F}} - \frac{\partial \Psi_{\text{total}}}{\partial \mathbb{H}} \cdot \dot{\mathbb{H}} - \sum_i \left[\frac{\partial \Psi_{\text{total}}}{\partial \mathbf{F}_i^v} : \dot{\mathbf{F}}_i^v\right] \\
&- \frac{\partial \Psi_{\text{total}}}{\partial \kappa_A} \cdot \dot{\kappa}_A - \frac{\partial \Psi_{\text{total}}}{\partial \mathbf{n}_A} \cdot \dot{\mathbf{n}}_A - \theta\,(\Gamma_0^{\kappa_A} + \Gamma_0^{\mathbf{n}_A}) = \\
&= \left(\mathbf{P} + p\mathbf{F}^{-\mathrm{T}} - \frac{\partial \Psi_{\text{total}}}{\partial \mathbf{F}}\right) : \dot{\mathbf{F}} + \left(-\mathbb{B} - \frac{\partial \Psi_{\text{total}}}{\partial \mathbb{H}}\right) \cdot \dot{\mathbb{H}} + \sum_i \left[-\frac{\partial \Psi_{\text{total}}}{\partial \mathbf{F}_i^v} : \dot{\mathbf{F}}_i^v\right] \\
&+ \left(-\theta\Gamma_0^{\kappa_A} - \frac{\partial \Psi_{\text{total}}}{\partial \kappa_A} \cdot \dot{\kappa}_A\right) + \left(-\theta\Gamma_0^{\mathbf{n}_A} - \frac{\partial \Psi_{\text{total}}}{\partial \mathbf{n}_A} \cdot \dot{\mathbf{n}}_A\right) \geq 0,
\end{aligned} \tag{25}$$

where θ is the absolute temperature, $\Gamma_0^{\kappa_A}$ and $\Gamma_0^{\mathbf{n}_A}$ represent entropic supply terms related to microstructural instabilities associated with the dynamic chain formation and p is a Lagrange multiplier to introduce an incompressibility constraint [15, 16]. Thereafter, through the application of the Coleman-Gurtin [13] or Coleman-Noll [17] procedures, we arrive to the following constitutive relations

$$\mathbf{P} = \frac{\partial \Psi_{\text{total}}}{\partial \mathbf{F}} - p\mathbf{F}^{-\mathrm{T}} \tag{26}$$

$$\mathbb{B} = -\frac{\partial \Psi_{\text{total}}}{\partial \mathbb{H}}. \tag{27}$$

The last two terms of Eq. 25 have to be greater or equal than zero. Similar to other research fields, such as in remodeling of biological tissues [18], we assume entropic sources to compensate the dynamic remodeling of the MREs. We assume a microstrutural remodeling driven by the supplied magnetic energy, reaching a state where the amount of energy needed to magnetize at the same intensity is minimum. Finally, the remaining term in the dissipation inequality, $\sum_i \left[-\frac{\partial \Psi_{\text{total}}}{\partial \mathbf{F}_i^v} : \dot{\mathbf{F}}_i^v\right] \geq 0$, establishes the necessary consistency conditions that each $\dot{\mathbf{F}}_i^v$ must satisfy.

1.5 Energy functions and constitutive equations

We define the strain energy potential per unit reference volume (without the free space term, Eq.16), $\Psi(\mathbf{F}, \mathbb{H}, \mathbf{F}_i^v, \kappa_A, \mathbf{n}_A)$, as a combination of magnetization, $\Psi_{\text{mag}}(\mathbf{F}, \mathbb{H}, \kappa_A, \mathbf{n}_A)$, isotropic,

$\Psi_{\text{iso}}(\mathbf{F},\mathbb{H},\mathbf{F}_i^v)$, and anisotropic contributions, $\Psi_{\text{ani}}(\mathbf{F},\mathbb{H},\mathbf{F}_i^v,\kappa_A,\ \mathbf{n}_A)$. Furthermore, the last two contributions can be further decomposed into equilibrium and non-equilibrium terms as

$$\begin{aligned}\hat{\Psi}(\mathbf{F},\mathbb{H},\mathbf{F}_i^v,\kappa_A,\mathbf{n}_A) &= \hat{\Psi}_{\text{mag}}(\mathbf{F},\mathbb{H},\kappa_A,\mathbf{n}_A) + \hat{\Psi}_{\text{iso}}^{\text{eq}}(\mathbf{F},\mathbb{H}) + \hat{\Psi}_{\text{iso}}^{\text{neq,st}}(\mathbf{F},\mathbb{H},\mathbf{F}_{\text{iso,st}}^v)\\ &+ \hat{\Psi}_{\text{iso}}^{\text{neq,lt}}(\mathbf{F},\mathbb{H},\mathbf{F}_{\text{iso,lt}}^v) + \hat{\Psi}_{\text{ani}}^{\text{eq}}(\mathbf{F},\mathbb{H},\kappa_A,\mathbf{n}_A) + \hat{\Psi}_{\text{ani}}^{\text{neq}}(\mathbf{F},\mathbb{H},\mathbf{F}_{\text{ani}}^v,\kappa_A,\mathbf{n}_A).\end{aligned} \tag{28}$$

It should be taken into account that, since the behavior of this family of MREs is so deeply dependent on the magnetic stimuli, the magnetic contributions will be included inside each of the terms of the expression above. A rheological representation of the model can be seen in Figure 5A in the Main Text.

The magnetization term is defined as

$$\hat{\Psi}_{\text{mag}}(\mathbf{F},\mathbb{H},\kappa_A,\mathbf{n}_A) = -\frac{\mu_0}{2}\chi^* I_7\,{}_2\mathcal{F}_1\left(\frac{1}{k_{mag}},\frac{2}{k_{mag}},1+\frac{2}{k_{mag}},-\left(\frac{\chi^*\sqrt{I_7}}{m_s}\right)^{k_{mag}}\right), \tag{29}$$

where

$$\chi^* = \chi\left[1+q_{mag}\tanh\left(p_{mag}I_9\right)\right], \tag{30}$$

χ and m_s are the magnetic susceptibility and the magnetic saturation, respectively, ${}_2\mathcal{F}_1$ denotes de hypergeometric function, k_{mag} is an exponent controlling how fast the material saturates, *tanh* refers to the hyperbolic tangent function and q_{mag} and p_{mag} are material parameters that estimate the impact on the magnetization due to the particles chains formation. Note that the last term in Eq. 30 introduces a time-dependent evolution of the material magnetization related to synergistic interactions at the microstructural level (as shown in experiments).

The equilibrium part of the isotropic term is defined by a Neo-Hookean energy potential, with μ_{iso}^{eq} being the shear modulus

$$\hat{\Psi}_{\text{iso}}^{\text{eq}}(\mathbf{F},\mathbb{H}) = \frac{\mu_{iso}^{eq}}{2}\left(I_1-3\right). \tag{31}$$

The non-equilibrium part is split into two principal branches that represent short-term and long-term relaxation mechanisms. The short-term potential is defined as

$$\hat{\Psi}_{\text{iso}}^{\text{neq,st}}(\mathbf{F},\mathbb{H},\mathbf{F}_{\text{iso,st}}^v) = \frac{\mu_{iso}^{neq,st}}{2}\left(1+\frac{q_0 I_7^{e,iso,st}}{1+\frac{q_0}{q_s}I_7^{e,iso,st}}\right)\left(I_1^{e,iso,st}-3\right), \tag{32}$$

with $I_1^{e,iso,st} = \mathrm{tr}\left(\left(\mathbf{F}_{\text{iso,st}}^v\right)^{-\mathrm{T}}\mathbf{F}^{\mathrm{T}}\mathbf{F}\left(\mathbf{F}_{\text{iso,st}}^v\right)^{-1}\right) = \mathrm{tr}\left(\mathbf{C}_{\text{iso}}^e\right)$. In the expression above, $\mu_{iso}^{neq,st}$ refers to the shear modulus of the short-term non-equilibrium part; $q_0 > 0$ describes the initial response of the magnetic coupling and $q_s > 0$ sets an upper saturation value to it [19]. On the other hand, the long-term non-equilibrium part of the isotropic contribution is defined as

$$\begin{aligned}\hat{\Psi}_{\text{iso}}^{\text{neq,lt}}(\mathbf{F},\mathbb{H},\mathbf{F}_{\text{iso,lt}}^v) &= \frac{\mu_{iso}^{neq,lt}}{2}\left(I_1^{e,iso,lt}-3\right)\\ &+\left[(1+\theta^{neq})f\left(I_4^{e,iso,lt}\right)+(1-\theta^{neq})f\left(I_8^{e,iso,lt}\right)-2f\left(I_7^{e,iso,lt}\right)\right],\end{aligned} \tag{33}$$

where $\mu_{iso}^{neq,lt}$ is the shear modulus of the long-term non-equilibrium part; θ^{neq} is a constant, and $f\left(I_i^j\right)$ has the following expression

$$f\left(I_i^j\right) = \frac{\beta_1^j \mu_0 m_s^2}{2\beta_2^j \chi} \log\left(1 + \frac{\beta_2^j \chi^2}{m_s^2} I_i\right) \quad \text{with} \quad j \in \{eq, neq\}, \tag{34}$$

where β_1^j, β_2^j are constants.
The last two terms of the strain energy potential, $\Psi_{\text{ani}}^{\text{eq}}$ and $\Psi_{\text{ani}}^{\text{neq}}$, are also based on the Neo-Hookean model using I_{10} instead of I_1. Moreover, I_9 is subtracted from I_{10} to ensure isochoric material response. The final expressions read as

$$\hat{\Psi}_{\text{ani}}^{\text{eq}}(\mathbf{F}, \mathbb{H}, \kappa_A, \mathbf{n}_A) = \tanh\left(w_0 I_9\right)\left[(1+\theta^{eq}) f\left(I_4\right) + (1-\theta^{eq}) f\left(I_8\right) - 2 f\left(I_7\right)\right] \tag{35}$$

$$\hat{\Psi}_{\text{ani}}^{\text{neq}}(\mathbf{F}, \mathbb{H}, \mathbf{F}_{\text{ani}}^v, \kappa_A, \mathbf{n}_A) = \frac{\mu_{ani}^{neq}}{2}\left(I_1^{e,ani} - 3\right) + \frac{\alpha_{ani}\mu_{ani}^{neq}}{2}\left(I_{10}^{e,ani} - I_9\right), \tag{36}$$

where μ_{ani}^{neq} is a material parameter with the same meaning as in the standard Neo-Hookean model, *tanh* refers to the hyperbolic tangent function, w_0, and θ^{eq} are constants, α_{ani} is a scaling factor and $f\left(I_i^j\right)$ is defined in Eq. (34).

1.6 Internal variables evolution

Finally, we need to define the evolution equations for the internal variables $\mathbf{F}_{\text{iso,st}}^v$, $\mathbf{F}_{\text{iso,lt}}^v$, $\mathbf{F}_{\text{ani}}^v$, κ_A and $\mathbf{n}_A$. The Bergstrom-Boyce model [20, 21, 22] and its generalization through the parallel network model [23], which is the one used in this work, exhibits the following viscous evolution

$$\dot{\mathbf{F}}_i^v = \dot{\gamma}_i^v (\mathbf{F}_i^e)^{-1} \frac{\text{dev}\boldsymbol{\sigma}_i}{\tau_i} \mathbf{F}_i^e \mathbf{F}_i^v \quad \text{with} \quad i \in \{(iso, st), (iso, lt), ani\}, \tag{37}$$

The viscous rate multiplier $\dot{\gamma}_i^v$ is defined as

$$\dot{\gamma}_i^v = \left(\frac{\tau_i}{\eta_i}\right)^{\frac{1}{m_i}}, \tag{38}$$

where τ_i, η_i and m_i are

$$\begin{aligned} \tau_i = ||\text{dev}\boldsymbol{\sigma}_i||_F, \quad \eta_i &= \eta_{0,i}\left(1 + \frac{s_{1,i}\sqrt{g_i\left(I_7\right)}}{\left(1 + \left(\frac{s_{1,i}}{s_{2,i}}\sqrt{g_i\left(I_7\right)}\right)^{p_i}\right)^{\frac{1}{p_i}}}\right) \quad \text{and} \\ m_i &= m_{0,i}\left(1 + \frac{z_{1,i}\sqrt{g_i\left(I_7\right)}}{\left(1 + \left(\frac{z_{1,i}}{z_{2,i}}\sqrt{g_i\left(I_7\right)}\right)^{r_i}\right)^{\frac{1}{r_i}}}\right)^{-1}. \end{aligned} \tag{39}$$

The operator $||\cdot||_F$ denotes the Frobenius norm [24]. Furthermore, it is important to clarify that $\boldsymbol{\sigma}_i$ is the non-equilibrium true stress related to either the isotropic or the anisotropic contributions, $\eta_{0,i}$ is the initial viscosity, $m_{0,i}$ is the initial exponent, and $s_{1,i}$, $s_{2,i}$, p_i, $z_{1,i}$, $z_{2,i}$ and r_i are material

constants [19]. The function inside η_i and m_i, i.e, g_i, depends on the nature of the associated contribution and has the following expression

$$g_i\left(I_7\right)=\begin{cases} I_7\left[1-\xi\left(\dfrac{\delta I_9}{\left(1+\left(\delta I_9\right)^{\upsilon}\right)^{\frac{1}{\upsilon}}}\right)\right], & \text{if } i\in\{ani\}\\ I_7, & \text{if } i\in\{(iso,st),(iso,lt)\},\end{cases} \tag{40}$$

where $\xi \in \{0, 1\}$ is a scaling factor controlling the influence of the anisotropy in the increased apparent viscosity, and δ and υ are material constants.
The evolution of κ_A is defined by the following expression:

$$\dot{\kappa}_A=-\eta_\kappa\left(\kappa_{limit}-\kappa_A\right)\left[\left(\kappa_{limit}-\kappa_A\right)^2-\left(\frac{1}{3}+b_\kappa\right)^2\right] \tag{41}$$

where κ_{limit} is

$$\kappa_{limit}\left(I_7\right)=\frac{1}{3}-\frac{\left(c_\kappa^2 I_7\right)^{p_\kappa}}{\left(1+3^{n_\kappa}\left(c_\kappa^2 I_7\right)^{p_\kappa n_\kappa}\right)^{\frac{1}{n_\kappa}}}, \tag{42}$$

Note that the value of κ_A oscillates between 1/3 and 0 depending on κ_{limit}. We introduce a dumping factor, η_κ, which controls the rate of κ_A to reach the equilibrium state, κ_{limit}, and a material parameter b_κ to change slightly the shape of $\dot{\kappa}_A$. c_κ, p_κ and n_κ are material parameters for tunning the saturation of κ_{limit}.
The generalization of this framework is achieved by defining the evolution of the direction to which particle chains tend to align, i.e., $\dot{\mathbf{n}}_A$. Motivated by previous works in biological remodelling [18, 25], we propose a reorientation equation with $\bar{\mathbb{H}}$ being the unit vector in the direction of $\mathbb{H}$ and to which $\mathbf{n}_A$ tends to be aligned:

$$\dot{\mathbf{n}}_A=\frac{1}{\tau_{n_A}}\left[\bar{\mathbb{H}}-\left(\bar{\mathbb{H}}\cdot\mathbf{n}_A\right)\mathbf{n}_A\right] \quad \text{and if} \quad \bar{\mathbb{H}}\cdot\mathbf{n}_A<0 \quad \text{then} \quad -\bar{\mathbb{H}}\mapsto\bar{\mathbb{H}}, \tag{43}$$

where τ_{n_A} is a relaxation parameter that controls the rate of the reorientation process.

1.7 Calibrated model parameters

Regarding the calibrated model parameters used in the simulations, Tables S1 to S5 report all of them. Note that the tags *long-term* and *short-term* may suggest that $\eta_{0,iso,st} < \eta_{0,iso,lt}$, drifting apart from the values displayed in such tables. However, given that the apparent viscosity of both branches grows differently with the external magnetic field, $\eta_{iso,st}$ has been seen to be smaller than $\eta_{iso,lt}$ during simulations when the imposed magnetic field is high. In addition, although τ_{n_A} was given a value of 1 s^{-1}, this parameter has not been calibrated, since the experimental setups used in this work only provide measurements on a single axis. To properly obtain this relaxation parameter, multiaxial rotating magnetic fields have to be imposed while measuring the evolution of the resulting magnetization vector.

χ[−]	q_{mag}[−]	p_{mag}[−]	k_{mag}[−]	$\mu_0 m_s$[T]	μ_{iso}^{eq}[kPa]
1.65	0.05	5e^5	2.75	0.665	0.73865

Table S1: Calibrated model parameters used in the simulations for the magnetization and the isotropic equilibrium branches. While χ and $\mu_0 m_s$ are directly obtained from magnetic hysteresis curves of the material; q_{mag}, p_{mag} and k_{mag} are obtained from magnetic relaxation data. The latter are used to account for the changes in the apparent magnetic susceptibility with the microstructural rearrangement of particles. Finally, μ_{iso}^{eq} determines the long-term and quasi-static mechanical response of the material and, thus, it is obtained from the purely mechanical relaxation curves. The units utilized for every listed parameter are included between square brackets.

$\mu_{iso}^{neq,st}$[kPa]	q_0[A^{-2}m^{-2}]	q_s[−]	$\eta_{0,iso,st}$[kPa · s]	$s_{1,iso,st}$[A^{-1}m^{-1}]	$s_{2,iso,st}$[−]
0.48485	1.885e^{-6}	50	115.511	0.1	1e^{-5}
$p_{iso,st}$[−]	$m_{0,iso,st}$[−]	$z_{1,iso,st}$[A^{-1}m^{-1}]	$z_{2,iso,st}$[−]	$r_{iso,st}$[−]	
2	1.493	10	1e^{-5}	4	

Table S2: Calibrated model parameters used in the simulations for the isotropic non-equilibrium short-term branch. The parameters $\mu_{iso}^{neq,st}$, $\eta_{0,iso,st}$ and $m_{0,iso,st}$ are used to introduce high strain rate and frequency dependencies and they are obtained from oscillatory shear tests without external magnetic field imposed. The parameters q_0 and q_s account for the increased stress, while $s_{1,iso,st}$, $s_{2,iso,st}$, $p_{iso,st}$, $z_{1,iso,st}$, $z_{2,iso,st}$ and $r_{iso,st}$ account for the changes in the apparent viscosity, when imposing fast magneto-mechanical actuation. The latter can be obtained from the oscillatory shear tests with magnetic actuation. The units utilized for every listed parameter are included between square brackets.

$\mu_{iso}^{neq,lt}$[kPa]	θ^{neq}[−]	β_1^{neq}[−]	β_2^{neq}[−]	$\eta_{0,iso,lt}$[kPa · s]	$s_{1,iso,lt}$[A^{-1}m^{-1}]
1449.265	−1	0.54	2.4	0.6835	0.05
$s_{2,iso,lt}$[−]	$p_{iso,lt}$[−]	$m_{0,iso,lt}$[−]	$z_{1,iso,lt}$[A^{-1}m^{-1}]	$z_{2,iso,lt}$[−]	$r_{iso,lt}$[−]
2.5e^3	2	0.781	10	1	4

Table S3: Calibrated model parameters used in the simulations for the isotropic non-equilibrium long-term branch. The parameters $\mu_{iso}^{neq,lt}$, $\eta_{0,iso,lt}$ and $m_{0,iso,lt}$ are used to introduce low strain rate and frequency dependencies and they are obtained from mechanical relaxation tests. θ^{neq}, β_1^{neq} and β_2^{neq} account for the initial sharp stress increments during the mechanically confined tests due to particles mobility when subjecting the sample to a fast magnetic actuation. Finally, the remaining parameters account for the changes in the apparent viscosity during magneto-mechanical relaxation tests. The units utilized for every listed parameter are included between square brackets.

$w_0[-]$	$\theta^{eq}[-]$	$\beta_1^{eq}[-]$	$\beta_2^{eq}[-]$	$\eta_\kappa[\mathrm{s}^{-1}]$	$b_\kappa[-]$	$c_\kappa[\mathrm{A}^{-1}\mathrm{m}^{-1}]$	$p_\kappa[-]$	$n_\kappa[-]$	$\tau_{n_A}[\mathrm{s}^{-1}]$
100	−0.5	0.132	0.05	2.5e^{-3}	0.12	2.6389e^{-6}	4	5	1

Table S4: Calibrated model parameters used in the simulations for the anisotropic equilibrium branch. The parameters w_0, θ^{eq}, β_1^{eq} and β_2^{eq} are used to determine the long-term and quasi-static magneto-mechanical response of the material in mechanically confined tests due to microstructual rearrangements of particles. On the other hand, the remaining parameters determine both, the transient formation and transient reorientation, of chain-like particles structures under magnetic actuation. The units utilized for every listed parameter are included between square brackets.

$\mu_{ani}^{neq}[\mathrm{kPa}]$	$\alpha_{ani}[-]$	$\eta_{0,ani}[\mathrm{kPa\cdot s}]$	$s_{1,ani}[\mathrm{A}^{-1}\mathrm{m}^{-1}]$	$s_{2,ani}[-]$	$p_{ani}[-]$	$m_{0,ani}[-]$
0.16873	1.75e^3	49.746	0.1	1e^{-5}	1	0.671
$z_{1,ani}[\mathrm{A}^{-1}\mathrm{m}^{-1}]$	$z_{2,ani}[-]$	$r_{ani}[-]$	$\xi[-]$	$\delta[-]$	$\upsilon[-]$	
10	1e^{-5}	4	1	6	6	

Table S5: Calibrated model parameters used in the simulations for the anisotropic non-equilibrium branch. The parameters μ_{ani}^{neq}, $\eta_{0,ani}$ and $m_{0,ani}$ are used to introduce medium strain rate and frequency dependencies and they are obtained from mechanical relaxation and oscillatory shear tests. On the other hand, α_{ani} introduces the double-bumping phenomenon exhibited during the magneto-mechanical relaxation tests. Finally, while $s_{1,ani}$, $s_{2,ani}$, p_{ani}, $z_{1,ani}$, $z_{2,ani}$ and r_{ani} account for the changes in the apparent viscosity due to the transient formation of chain-like particles structures under magnetic actuation, the parameters ξ, δ and υ introduce a viscosity recovery mechanism activated when these chain-like particles structures have been already formed, i.e., the particles are no longer moving. The units utilized for every listed parameter are included between square brackets.

2 Supplementary Results

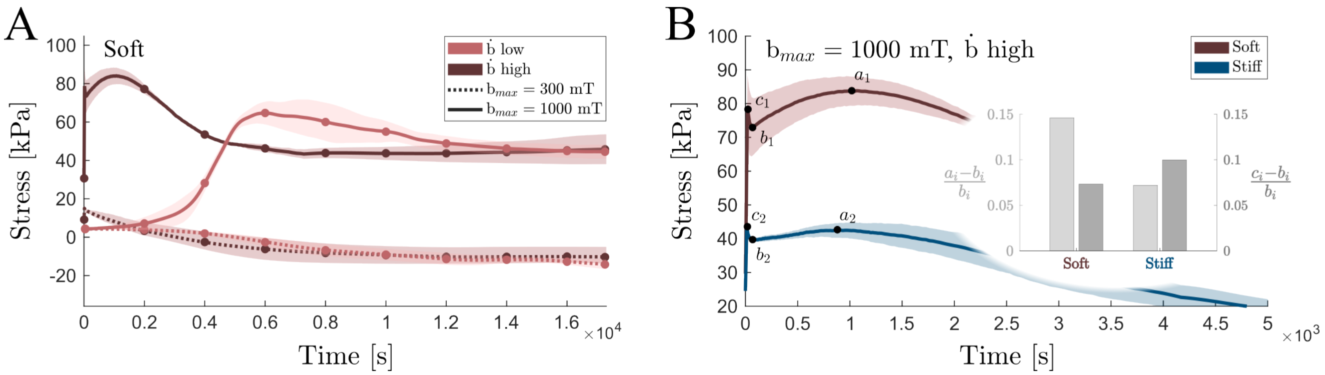

Figure S1: **Additional magnetic relaxation tests on mechanically confined MRE samples.** **(A)** Comparison of stress relaxation tests applying external magnetic ramps combining different magnetic field magnitudes (300 and 1000 mT) and loading rates ($\dot{b}_{low}$ = 0.2 mT·s^{-1} and $\dot{b}_{high}$ = 1000 mT·s^{-1}) on soft sMREs samples (30% CIP volume fraction, diameter = 18 mm). **(B)** Detail of the comparison of stress relaxation tests applying an external magnetic ramp reaching 1000 mT in 1 s, measuring the time evolution of the axial stress on mechanically confined soft and stiff sMREs samples (30% CIP volume fraction, diameter = 18 mm).

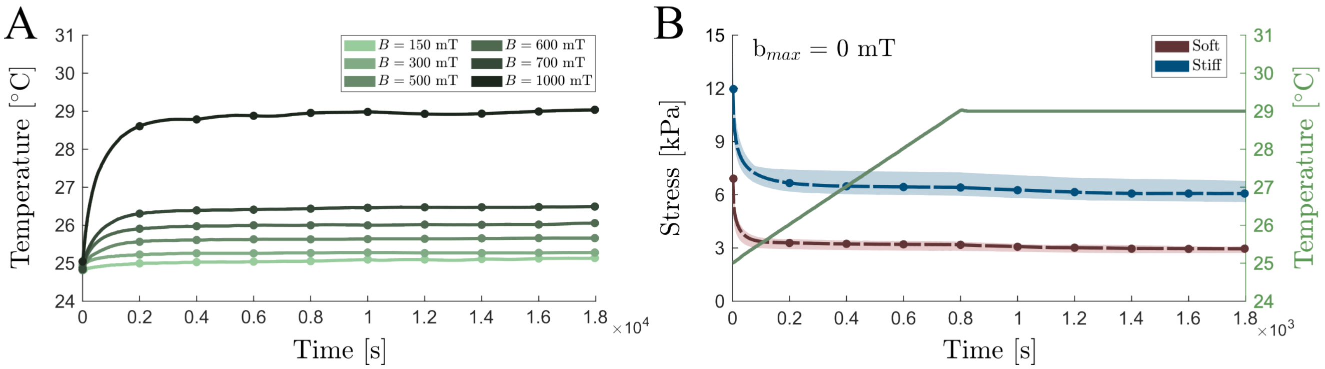

Figure S2: **Temperature increase in MRE samples due to magnetic actuation and thermo-mechanical effects.** **(A)** Temperature evolution over time on MRE samples subjected to magnetic ramps of different magnitudes. These tests used soft sMREs samples (30% CIP volume fraction, diameter = 18 mm). **(B)** Mechanical relaxation tests on soft and stiff MRE samples, controlling the temperature evolution over time so that it simulates the thermal effects introduced by magnetic actuation. No relevant effects are observed.

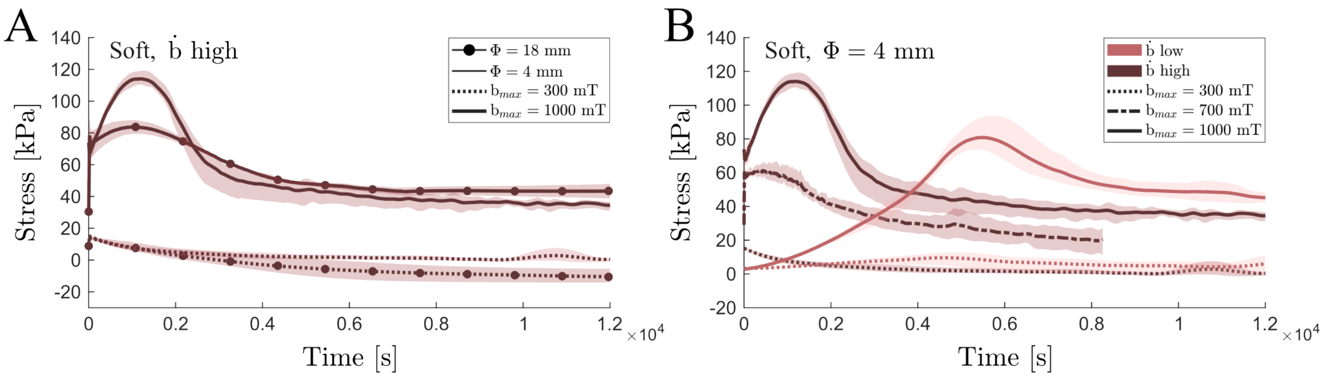

Figure S3: **Structural effects on magnetically induced relaxation processes, and dependencies on magnetic actuation characteristics in soft MRE samples.** **(A)** Comparison of stress relaxation tests applying external magnetic ramps at a high loading rate (1000 mT·s^{-1}) combining different magnetic field magnitudes (300 and 1000 mT) and sample diameters (4 mm and 18 mm) on soft ($\sim$ 1 kPa) sMREs samples of 1 mm height (30% CIP volume fraction). **(C)** Comparison of stress relaxation tests applying external magnetic ramps combining different magnetic field magnitudes (300, 700 and 1000 mT) and loading rates ($\dot{b}_{low} = 0.2$ mT·s^{-1} and $\dot{b}_{high} = 1000$ mT·s^{-1}) on soft sMREs samples of 4 mm diameter.

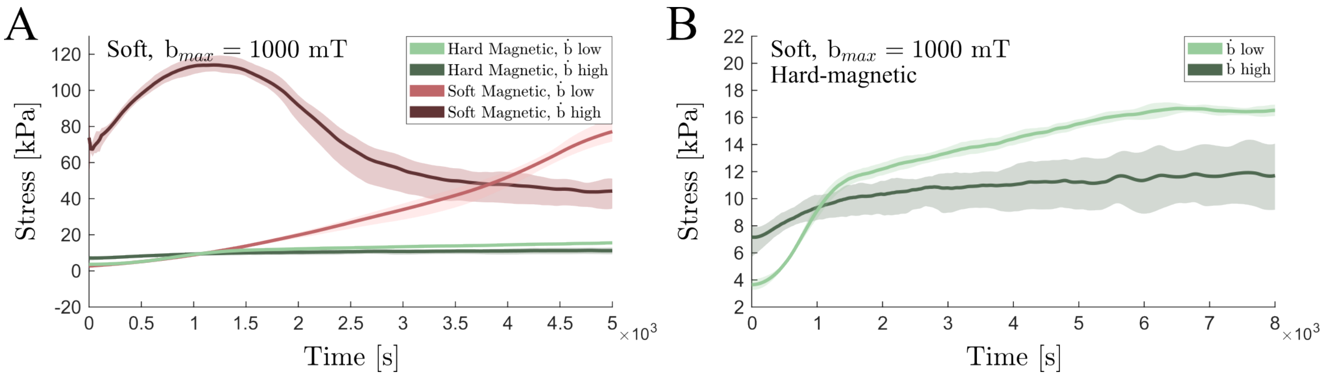

Figure S4: **Magneto-mechanical relaxation tests on soft MRE samples. (A)** Comparison of stress relaxation tests applying external magnetic ramps of 1000 mT at different loading rates ($\dot{b}_{low} = 0.2$ mT·s^{-1} and $\dot{b}_{high} = 1000$ mT·s^{-1}) on soft (~ 1 kPa) sMRE and hMRE samples. **(B)** Detail of the responses of hMRE sample tests presented in panel (A).

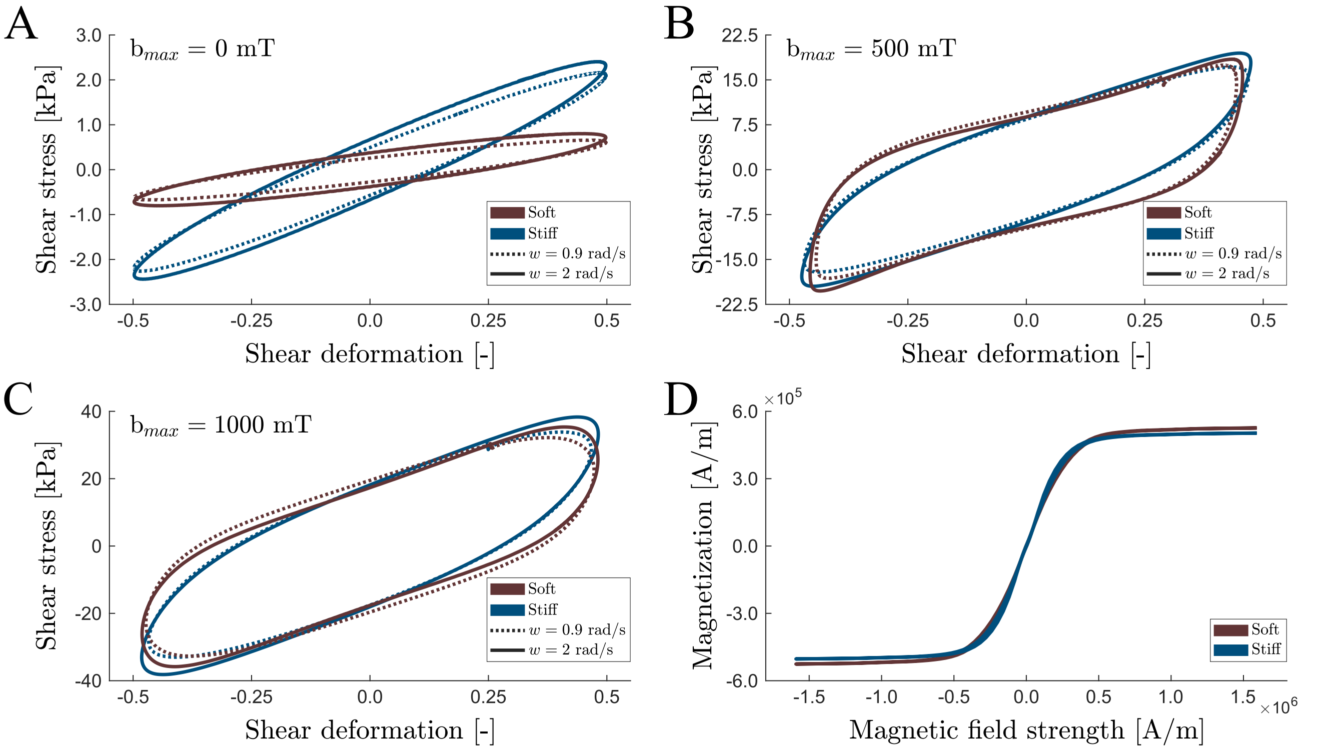

Figure S5: **Mechanical, magnetic and magneto-mechanical characterization of sMRE samples.** Cyclic shear loading under two different rates on soft (~ 1 kPa) and stiff (~ 10 kPa) sMRE samples for: **(A)** null external magnetic field, **(B)** an external magnetic field of 500 mT, and **(C)** an external magnetic field of 1000 mT.**(D)** Experimental data for the magnetization response of both soft and stiff sMRE samples.

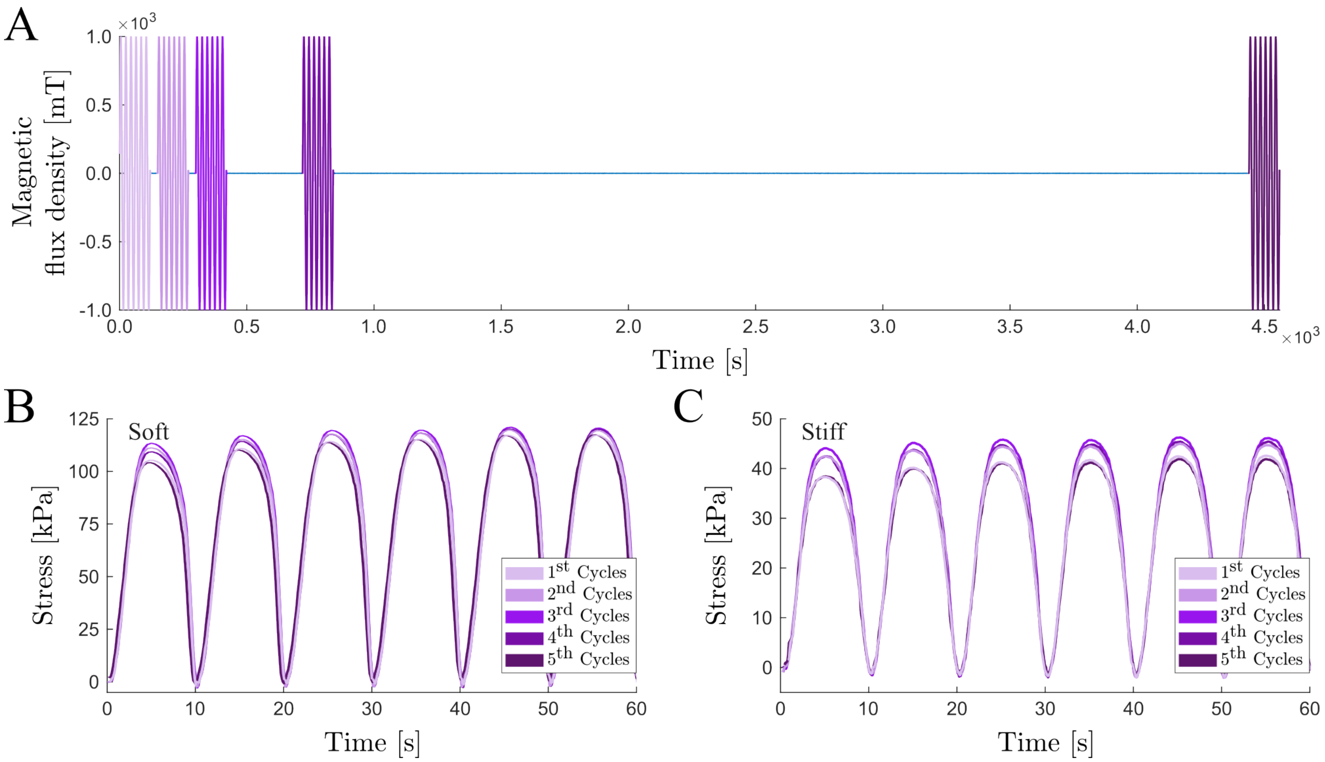

Figure S6: **Additional experiments on soft sMRE samples exposed to cyclic magnetic actuation under mechanically confined conditions.** The history dependent stress response in sMRE samples is experimentally tested imposing magnetic actuation cycles with different relaxation periods between them **(A)**. Stress response during the first 60 s of the different magnetic cycles on **(B)** soft (∼ 1 kPa) and **(C)** stiff (∼ 10 kPa) sMRE samples.

References

[1] E. H. Lee, *Journal of Applied Mechanics* **1969**, *36*, 1 1.

[2] P. Saxena, M. Hossain, P. Steinmann, *International Journal of Solids and Structures* **2013**, *50*, 24 3886.

[3] A. Dorfmann, R. Ogden, *Acta Mechanica* **2004**, *167* 13.

[4] D. Mukherjee, L. Bodelot, K. Danas, *International Journal of Non-Linear Mechanics* **2020**, *120* 103380.

[5] D. Garcia-Gonzalez, M. Hossain, *Extreme Mechanics Letters* **2021**, *48* 101382.

[6] D. Mukherjee, M. Rambausek, K. Danas, *Journal of the Mechanics and Physics of Solids* **2021**, *151* 104361.

[7] K. Danas, P. M. Reis, *Journal of the Mechanics and Physics of Solids* **2024**, *191* 105764.

[8] A. Dorfmann, R. Ogden, *Quarterly Journal of Mechanics and Applied Mathematics* **2004**, *57*, 4 599.

[9] G. Brovko, *Mathematical and Computational Applications* **2019**, *24* 79.

[10] Q.-S. Zheng, *Applied Mechanics Reviews* **1994**, *47*, 11 545.

[11] A. Spencer, In A. C. ERINGEN, editor, *Mathematics*, 239–353. Academic Press, ISBN 978-0-12-240801-4, **1971**, URL https://www.sciencedirect.com/science/article/pii/B978012240801450008X.

[12] G. Holzapfel, *Nonlinear Solid Mechanics: A Continuum Approach for Engineering*, John Wiley & Sons, New York, **2000**.

[13] B. D. Coleman, M. E. Gurtin, *The journal of chemical physics* **1967**, *47*, 2 597.

[14] G. A. Maugin, *Continuum mechanics of electromagnetic solids*, Elsevier, **2013**.

[15] C. Kadapa, M. Hossain, *Mechanics of Advanced Materials and Structures* **2022**, *29*, 2 267.

[16] E. A. de Souza Neto, D. Peric, D. R. Owen, *Computational methods for plasticity: theory and applications*, John Wiley & Sons, **2011**.

[17] B. D. Coleman, W. Noll, *Archive for Rational Mechanics and Analysis* **1963**, *13* 167.

[18] A. Menzel, *Biomechanics and modeling in mechanobiology* **2005**, *3*, 3 147—171.

[19] K. Danas, M. Nakano, G. Sebald, *Mechanics of Materials* **2025**, *200* 105187.

[20] J. Bergström, M. Boyce, *Journal of the Mechanics and Physics of Solids* **1998**, *46*, 5 931 .

[21] J. Bergström, M. Boyce, *Mechanics of Materials* **2001**, *33* 523.

[22] J. S. Bergstrom, M. C. Boyce, *Rubber Chemistry and Technology* **1999**, *72*, 4 633.

[23] J. Bergstrom, J. Bischoff, *International Journal of Structural Changes in Solids* **2010**, *2* 31.

[24] J. Bergstrom, *Mechanics of Solid Polymers: Theory and Computational Modeling*, William Andrew, Elsevier, London, UK, 1 edition, **2015**.

[25] A. Melnik, A. Goriely, *Mathematics and Mechanics of Solids* **2013**, *18* 634.
